# Herbal medicine, *Banxia-xiexin tang*, for functional dyspepsia: a systematic review and meta-analysis

**DOI:** 10.3389/fphar.2023.1130257

**Published:** 2023-05-19

**Authors:** Keumji Kim, Seok-Jae Ko, Soo Ho Cho, Jinsung Kim, Jae-Woo Park

**Affiliations:** ^1^ Department of Internal Korean Medicine, Kyung Hee University Hospital at Gangdong, Seoul, Republic of Korea; ^2^ Department of Clinical Korean Medicine, Graduate School of Kyung Hee University, Seoul, Republic of Korea; ^3^ Department of Gastroenterology, College of Korean Medicine, Kyung Hee University, Seoul, Republic of Korea

**Keywords:** functional dyspepsia, Banxia-xiexin tang, herbal medicine, meta-analysis, systematic review

## Abstract

**Background:** The demand for complementary and alternative medicine for the management of functional dyspepsia (FD) is increasing due to the insufficient efficacy of conventional treatment options. In Asia, the Chinese herbal medicine formula *Banxia-xiexin tang* (BXT) has been used to treat FD.

**Methods:** We searched 11 digital medical databases on 1 September 2021. Randomized controlled trials (RCTs) that investigated the efficacy of BXT or combination therapy (BXT plus Western medicines) for FD were selected. The outcome parameters were total clinical efficacy rate (TCE), motilin level, symptom checklist-90-revised (SCL-90-R), and visual analog scale (VAS) for dyspepsia and adverse events. Cochrane risk of bias tool 2.0 (RoB 2) was used for the quality assessment of included studies.

**Results:** The meta-analysis comprised 57 RCTs with 5,525 participants. BXT was more efficacious, with a higher TCE than Western medicine. Combination therapy (BXT plus Western medicine) also resulted in a higher TCE than Western medicine. Combination therapy improved motilin levels and psychological symptoms to a greater extent than Western medicine, evidenced by a higher SCL-90-R score. However, no significant difference in VAS scores was observed between the BXT and placebo groups. BXT and combination therapy were associated with fewer adverse events than Western medicine or placebo.

**Conclusion:** Our findings suggest that BXT and its combination therapy may be an effective and safe alternative treatment for FD. More RCTs with better methodologies are required to strengthen this evidence.

**Systematic Review Registration:** [https://www.crd.york.ac.uk/prospero/display_record.php?ID=CRD42019123285], identifier [CRD42019123285].

## 1 Introduction

Functional dyspepsia (FD) is a common clinical disorder characterized by dyspeptic symptoms, such as early satiation, postprandial fullness, epigastric pain, or burning, that persist despite routine medical evaluations (Tack et al., 2006). The global prevalence of FD ranges from 11% to 29.12% (Mahadeva and Goh, 2006). A recent study reported that FD was the most common gastroduodenal disease, with pooled prevalence rates of 7.2% on the Internet and 4.8%, on a household survey (Sperber et al., 2020). The pathophysiology of FD is multifactorial and has not been completely explained (Stanghellini et al., 2016). Gastric motility, sensory disorders, mucosal permeability, low-grade immune activation, dysregulation of the gut-brain axis, and environmental exposure are all potential causative factors of FD (Vanheel and Farré, 2013). *Helicobacter pylori* (*Helicobacter pylori*) eradication, prokinetic (PK) agents, acid suppressants, and central neuromodulators are conventional treatments for FD (Moayyedi et al., 2017). However, an incomplete understanding of the pathophysiology of FD makes the treatment difficult (Ford et al., 2020). The need for complementary and alternative treatments, including herbal medicines, is growing because conventional treatments are less efficacious (Suzuki et al., 2009).

*Banxia-xiexin tang* (BXT; 半夏瀉心湯; Banha-sasim tang in traditional Korean medicine; Hange-shashin-to in Kampo medicine) is an herbal medicine formula that comprises 7 botanical drugs and originally recorded in the old Chinese literature “Shan han za bing lin (傷寒雜病論)”. It has been used in various versions by adding or subtracting botanical drugs. In Korea, BXT is produced in granules according to Korean Good Manufacturing Practice under the regulation of the Ministry of Food and Drug Safety. BXT (1/3 pack dose of the formula [貼] is as follows: Pinellia ternata (Thunb.) Makino [Araceae; Pinellia ternata rhizoma] 1.67g, Panax ginseng C.A.Mey. [Araliaceae; Panax ginseng root] 1.00g, Zingiber officinale Roscoe [Zingiberaceae; Zingiber officinale rhizoma] 0.83g, Coptis chinensis Franch. [Ranunculaceae; Coptis chinensis rhizoma] 0.33g, Scutellaria baicalensis Georgi [Lamiaceae; Scutellaria baicalensis root] 1.00g, Ziziphus jujuba Mill. [Rhamnaceae; Zizyphus jujuba fruit] 1.00 g) is extracted (0.91 g) in boiling water and mixed with lactose (0.52 g) and starch (1.57 g) then given 3 g of granules (Park et al., 2010). In traditional Chinese medicine, this formula has been administered to treat “epigastric stuffiness,” a symptom of FD (Park et al., 2010). In addition to FD, BXT has been administered to treat a wide range of gastrointestinal (GI) diseases, including gastroesophageal reflux disease (GERD), acute gastroenteritis, chronic gastritis, peptic ulcers, and ulcerative colitis (Ji et al., 2017). A systematic review reported that modified BXT is an efficacious treatment option for GERD (Dai et al., 2017), and another meta-analysis reported that BXT is more efficacious than Western medicine for treating diabetic gastroparesis (Tian et al., 2013). BXT and combination therapy (BXT plus Western medicine) had a better effect on ulcerative colitis, according to another meta-analysis (Zhu et al., 2016). One systematic review reported that combination therapy (BXT plus Western medicine) was more efficacious than Western medicine alone, in treating peptic ulcers (Chen et al., 2014).

Systematic reviews that investigated the effects of BXT on FD have been published earlier. However, some studies only compared BXT and Western medicine and did not investigate the effects of combination therapy (BXT plus Western medicine) (Gan et al., 2014; Zhang, 2015; Li and Li, 2016; Hu et al., 2020). One review was based on a Chinese database (Li and Li, 2016). In another meta-analysis, it was difficult to focus on the effects of BXT because of the high heterogeneity of the experimental group (Zhang, 2015). Consequently, limited evidence supports BXT as an efficacious treatment option for FD. Therefore, this review aimed to systematically investigate the efficacy and safety of BXT and combination therapy (BXT plus Western medicine) in the treatment of FD.

## 2 Methods

### 2.1 Protocol and registration

The study protocol was registered in the International Prospective Register of Systematic Reviews. The registration number is CRD42019123285 (Ko et al., 2019). This systematic review was conducted in accordance with the Preferred Reporting Items for Systematic Reviews and Meta-analyses guidelines (Liberati et al., 2009).

### 2.2 Inclusion and exclusion criteria

#### 2.2.1 Types of studies

This systematic review included randomized controlled trials (RCTs) and quasi-RCTs.

#### 2.2.2 Types of participants

This systemic review included patients diagnosed with FD according to the ROME criteria. No limitations were observed on the participant’s age, sex, or ethnicity. The ROME criteria were used as the diagnostic criteria for screening functional GI disorders (FGID). The ROME IV criteria were finalized in 2016, after being announced in 1992 and after several revisions. The inclusions of studies published before adopting the ROME I criteria in 1992 were decided by the consensus of two reviewers (KK and SC), who assessed whether the diagnostic criteria were compatible with the ROME I criteria. Patients with dyspepsia caused by drugs or secondary pathologies (e.g., GERD and irritable bowel syndrome) were excluded.

#### 2.2.3 Types of interventions

This systematic review included studies on BXT, modified BXT, and combination therapy (BXT and Western medicine). Modified BXT is BXT with additional medicinal botanical drugs, for example, *Chaizhi* BXT (CZBXT; 柴枳半夏瀉心湯; BXT added to Bupleurum falcatum L. [Apiaceae; Bupleurum falcatum root] and Citrus trifoliata L. [Rutaceae; Citrus trifoliata immature fruit]. We ruled out modified BXT if the botanical drugs added to BXT resulted in another herbal medicine formula. Combinations of BXT and other treatments in complementary and alternative medicine, such as acupuncture and moxibustion, were excluded. The following medication groups were compared in this study: a) the BXT and modified BXT groups with Western medicine (such as PK agents and proton pump inhibitors [PPIs]); b) the BXT group with placebo (same taste, shape, color, and odor as BXT) group; and c) combination therapy (BXT plus Western medicine) group with Western medicine group.

#### 2.2.4 Types of outcome measures

The total clinical efficacy rate (TCE) was the primary outcome. TCE is the percentage of patients who responded to treatment (Tang, 2015; Zou, 2015). The patients’ improvements to the interventions were graded into three or four levels after treatment, and TCE was calculated as the total number of improved patients. TCE with three levels includes “cured” or “excellently improved,” “improved,” and “not improved,” while TCE with four levels includes “cured,” “excellently improved,” “improved,” and “not improved.” Although the number of evaluation levels differs, it is the same standard in that the ratio of numbers excluding ‘not improved’ from the total is calculated.

The secondary outcomes included motilin levels, symptom checklist-90-revised (SCL-90-R) score, visual analog scale (VAS) pain scores, and adverse events. Motilin, a GI hormone, induces GI motor activity (Naito et al., 2002), and increased motilin secretion might improve GI mobility. The SCL-90-R is a self-rating scale used to evaluate psychological symptoms, and a higher SCL-90-R score indicates greater psychiatric distress (Faramarzi et al., 2014). The VAS was used to quantify the degree of indigestion symptoms felt by the patient, and the number of side effects was compared.

### 2.3 Search strategy

A literature search was conducted using Medline (via PubMed), Cochrane Central Register of Controlled Trials, EMBASE, and Allied and Complementary Medicine Databases on 1 September 2021. Medical databases in Korea, including the National Digital Science Library, Korean Medical Database, Korean Studies Information Service System, KoreaMed, and Oriental Medicine Advanced Searching Integrated System, were also searched. Additionally, other Asian databases, including China National Knowledge Infrastructure Database in Chinese and Citation Information by Nii in Japanese, were searched.

The search terms were composed of disease- and intervention-related terms. Disease-related terms included “indigestion,” “dyspepsia,” “discomfort,” “disturbance,” “pain,” “dysfunction,” “intestine,” “stomach,” and “gut.” Terms such as “Banha sasim,” “Banxia xiexin,” “Hange shashin,” “herbal medicine,” and “botanical” were used as intervention-related terms. Language and publication dates were not restricted.

### 2.4 Selection and data extraction

Two authors (KK and SC) independently screened the studies to evaluate their eligibility for inclusion. The selected paper’s titles, abstracts, and full texts were screened. Endnote X9 (Clarivate Analytics, Philadelphia, PA, United States) was used to manage the search results. Furthermore, these authors independently extracted data from the studies and filled out a standard data extraction form. The form included information on the studies, such as the first authors, titles, publication years, journals, research design, interventions, sample sizes, treatment period, and outcomes. Disagreements between the two reviewers (KK and SC) were resolved through discussion. If the two reviewers could not reach an agreement, an arbiter (SK) intervened and resolved the discrepancies.

### 2.5 Quality assessment

Two reviewers (KK and SC) independently evaluated the risk of bias (RoB) using the Cochrane RoB tool 2.0 with the following items: a) bias arising from the randomization process, b) bias due to deviations from intended interventions, c) bias due to missing outcome data, d) bias in the measurement of the outcome, e) bias in the selection of the reported result, and f) overall bias. The assessment results were divided into three categories: low, high, and some concerns. All disagreements between the two evaluators (KK and SC) were discussed. When needed, the arbiter (SK) intervened and resolved the disagreement.

### 2.6 Data analysis and synthesis

Review Manager (V5.3 Copenhagen: The Nordic Cochrane Center, The Cochrane Collaboration, 2014) was used for data analysis. Dichotomous data were assessed for relative risk (RR) with a 95% confidence interval (CI), and continuous data were evaluated using the mean difference (MD) with 95% CI. A random-effects model was used for meta-analysis. TCE, the only ordinal scale in this study, was treated as a dichotomous scale by dividing it into “not improved” and “improved.” To judge the heterogeneity of the selected studies, we used the chi-squared (χ^2^) test and *I*-squared statistics (*I* ^2^). A *p*-value < 0.1 and *I* ^2^ ≥ 50 indicated substantial heterogeneity. Subgroup analyses were performed. The subgroups were formed based on the type of Western medicine administered. A funnel plot was used to present small-study effects or publication bias.

### 2.7 Level of evidence

The level of evidence was examined using the Grading of Recommendations, Assessment, Development, and Evaluation approach. The level of evidence was classified as high, moderate, low, or very low. The evaluation was performed for domains such as the RoB, imprecision, inconsistency, and indirectness.

## 3 Results

### 3.1 Selection of study

**FIGURE 1:**
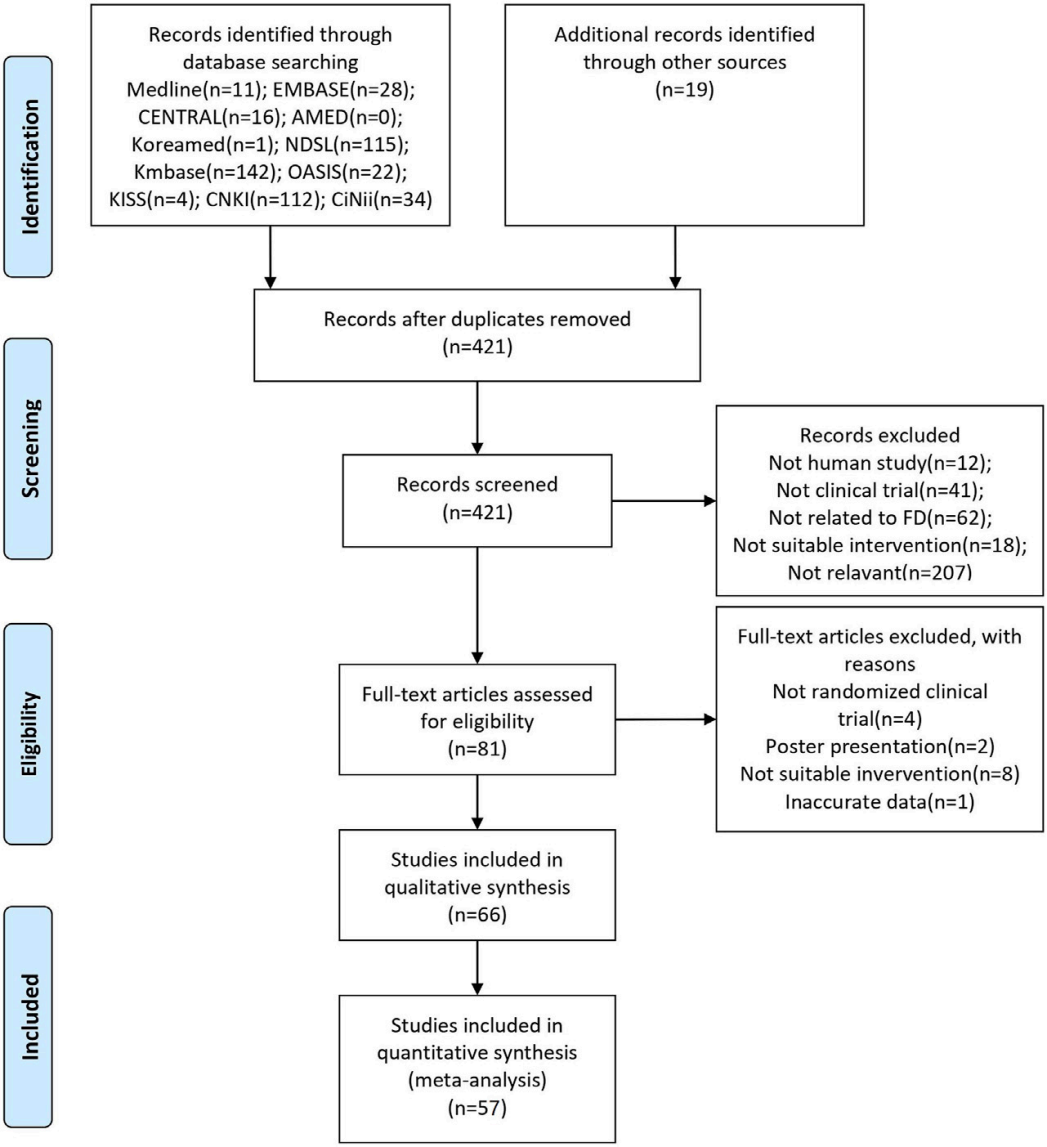
PRISMA flow chart of the selection process. EMBASE: Excerpta Medica Database; CENTRAL: Cochrane Central Register of Controlled Trials; AMED: Allied and Complementary Medicine Database; NDSL: National Digital Science Library; Kmbase: Korean Medical Database; OASIS: Oriental Medicine Advanced Searching Integrated System; KISS: Korean Studies Information Service System; CNKI: China National Knowledge Infrastructure Database; CiNii: Citation Information by Nii; FD: Functional dyspepsia.

The initial search identified 504 studies, of which 83 duplicates were excluded. After screening the titles and abstracts, 340 studies were excluded. Fifteen studies were excluded because 14 did not meet the inclusion criteria, and one presented inaccurate data. Finally, 66 studies were included, and 57 were selected for the meta-analysis (Figure 1).

### 3.2 Characteristics of included studies

We included 66 studies published between 2001 and 2021. All articles were RCTs and parallel-design studies. Four studies were written in English and 62 in Chinese. Three randomized trials were conducted in Korea and 63 in China. BXT was compared with Western medicine in 51 RCTs. In 10 articles, combination therapy (BXT plus Western medicine), and Western medicine were compared. BXT was also compared with a placebo in five RCTs. The total number of patients included in the meta-analysis was 5,615. In each study, 31–200 patients were included. Table 1 shows a summary of the analyzed RCTs.

**TABLE 1:** Characteristics of included studies.

Study ID		Language	Study design	Intervention (n)	Control (n)		Duration	Outcome	Results
Dong et al. (2017)		Chinese	Parallel	BXT (49)	Domperidone (49)		4 weeks	① TCE	① 91.84% vs. 79.59% (*p* < 0.05)
								② Symptom score	② belching
									0.64 ± 0.19 vs. 0.80 ± 0.20 (*p* < 0.05) nausea
									0.78 ± 0.24 vs. 0.83 ± 0.41 (*p* < 0.05) epigastric bloating
									0.99 ± 0.24 vs. 1.43 ± 0.43 (*p* < 0.05) epigastric pain
									0.78 ± 0.33 vs. 1.12 ± 0.46 (*p* < 0.05) epigastric burning
									0.60 ± 0.18 vs. 0.95 ± 0.28 (*p* < 0.01)
								③ Gastric MMCs	③ 20.88 ± 1.24 vs. 26.09 ± 5.31 (*p* < 0.05)
								④ MTL	④ 410.12 ± 79.94 vs. 386.42 ± 81.62 (*p* < 0.05)
								⑤ GE T1/2	⑤ 25.86 ± 4.96 vs. 32.11 ± 5.02 (*p* < 0.05)
Feng et al. (2015)		Chinese	Parallel	BXT (53)	Domperidone (53)		4 weeks	① TCE	① 92.45% vs. 86.79% (*p* < 0.05)
								② Symptom score	② The BXT group was better than the control group (*p* < 0.05)
								③ Serum SP	③ 36.68 ± 11.89 vs. 44.28 ± 2.38 (*p* < 0.05)
								④ CGRP	④ 1.15 ± 0.64 vs. 1.21 ± 0.20 (*p* < 0.05)
He (2007)		Chinese	Parallel	CZBXT (42)	Domperidone (40)		4 weeks	① TCE	① 85.7% vs. 65.0% (*p* < 0.05)
Hu et al. (2006)		Chinese	Parallel	BXT (30)	Domperidone (30)		2 weeks	① TCE	① 90.0% vs. 86.7% (*p* > 0.05)
								② TCM SS	② 5.23 ± 3.28 vs. 7.03 ± 3.11 (*p* < 0.05)
								③ MTL	③ 312.02 ± 29.49 vs. 311.24 ± 31.77 (*p* > 0.05)
Jin et al. (2004)		Chinese	Parallel	BXT (90)	Domperidone (90)		4 weeks	① TCE	① 90.00% vs. 88.88% (*p* > 0.05)
								② Symptom score	② epigastric fullness
									2.74 ± 1.26 vs. 2.39 ± 1.14 (*p* > 0.05) nausea and vomiting
									1.01 ± 1.24 vs. 1.83 ± 1.54 (*p* < 0.05) belching
									2.55 ± 0.67 vs. 3.72 ± 0.65 (*p* < 0.05) early satiety
									1.81 ± 1.30 vs. 1.92 ± 1.25 (*p* > 0.05) loose stool
									1.54 ± 1.43 vs. 2.75 ± 1.74 (*p* < 0.05)
								③ GE T1/2	③ 20.74 ± 7.42 vs. 21.01 ± 7.25 (*p* > 0.05)
								④ EGG	④ frequency primary (FP)
									2.85 ± 0.74 vs. 2.50 ± 0.51 (*p* < 0.05) frequency zero (FZ)
									2.94 ± 0.68 vs. 2.67 ± 0.63 (*p* < 0.05) frequency caliz (FC)
									2.63 ± 0.85 vs. 2.30 ± 0.91 (*p* < 0.05)
Li (2015)		Chinese	Parallel	BXT (45)	Domperidone (43)		4 weeks	① TCE	① 89% vs. 78% (*p* < 0.05)
								② Symptom score	② total score
									9.73 ± 3.95 vs. 14.25 ± 4.02 (*p* < 0.05) postprandial fullness
									2.42 ± 1.22 vs. 3.92 ± 1.53 (*p* < 0.05) early satiety
									2.18 ± 1.04 vs. 3.51 ± 1.20 (*p* < 0.05) epigastric pain
									1.52 ± 0.73 vs. 2.44 ± 0.69 (*p* > 0.05) epigastric burning
									2.54 ± 0.67 vs. 2.89 ± 0.95 (*p* < 0.05)
Liu 2020		Chinese	Parallel	BXT (60)	Domperidone (60)		4 weeks	① TCE	① 91.7% vs. 75% (*p* < 0.05)
								② Symptom score	② epigastric fullness
									1.88 ± 0.53 vs.2.43 ± 0.89 (*p* < 0.05) regurgitation
									2.11 ± 0.41 vs. 2.94 ± 0.83 (*p* < 0.05) coldness
									1.76 ± 0.56 vs. 2.34 ± 0.62 (*p* < 0.05) bitter taste and dry mouth
									1.15 ± 0.28 vs. 2.00 ± 0.32 (*p* < 0.05) loose stool
									1.56 ± 0.33 vs. 2.23 ± 0.42 (*p* < 0.05)
								③ Gastric mobility	③ normal slow wave
									4.34 ± 2.56 vs.3.34 ± 2.40 (*p* < 0.05) amplitude (long diameter)
									1.91 ± 0.20 vs. 0.67 ± 0.10 (*p* < 0.05) amplitude (short diameter)
									0.98 ± 0.20 vs. 0.67 ± 0.10 (*p* < 0.05) gastric half emptying time
									52.32 ± 16.32 vs. 48.46 ± 12.14 (*p* < 0.05) gastric emptying time
									92.34 ± 13.34 vs.98.51 ± 11.21 (*p* < 0.05)
Qiu (2011)		Chinese	Parallel	BXT (49)	Domperidone (49)		4 weeks	① TCE	① 89.8% vs. 83.7% (*p* < 0.05)
								② TCM SS	② 6.75 ± 2.66 vs. 8.02 ± 2.90 (*p* < 0.05)
Ren (2015)		Chinese	Parallel	BXT (45)	Domperidone (45)		4 weeks	① TCE	① 93.3% vs. 57.8% (*p* < 0.05)
								② Symptom score	② 0.84 ± 0.19 vs. 1.97 ± 0.24 (*p* < 0.05)
Tian (2018)		Chinese	Parallel	BXT (40)	Domperidone (40)		1 month		① 97.5% vs. 77.5% (*p* < 0.05)
									② physiological skills
								① TCE	93.30 ± 4.13 vs. 81.35 ± 4.15(*p* < 0.01) somatalgia
								② QOL	94.36 ± 4.17 vs. 80.44 ± 4.23(*p* < 0.01) physiological function
									95.48 ± 4.33 vs. 82.59 ± 4.38(*p* < 0.05)
Wu (2008)		Chinese	Parallel	BXT (50)	Domperidone (40)		2 weeks	① TCE	① 94.0% vs. 62.5% (*p* < 0.01)
Yu and Yang (2010)		Chinese	Parallel	BXT (90)	Domperidone (45)		1 month	① TCE	① 90% vs. 55.6% (*p* < 0.05)
								② Effective rate	② epigastric pain
									90.0% vs. 64.0% (*p* < 0.05) bloating
									91.3% vs. 61.0% (*p* < 0.05) early satiety
									82.9% vs. 65.8% (*p* < 0.05) belching
									89.4% vs. 68.8% (*p* < 0.05) acid reflux
									81.6% vs. 61.1% (*p* < 0.05) nausea
									84.2% vs. 60.0% (*p* < 0.05)
								③ Symptom score	③ 9.96 ± 3.52 vs. 9.22 ± 2.91 (*p* < 0.01)
Zhao and Song (2011)		Chinese	Parallel	BXT (48)	Domperidone (48)		4 weeks	① TCE	① 89.6% vs. 83.3% (*p* < 0.05)
								② TCM SS	② 6.17 ± 3.11 vs. 7.89 ± 3.90 (*p* < 0.05)
Zhao and Su (2017)		Chinese	Parallel	BXT (31)	Domperidone (30)		4 weeks	① TCE	① 90.32% vs. 76.67% (*p* < 0.05)
								② TCM SS	② The BXT group was better than the control group in 5 items (*p* < 0.05), and there was no difference in 2 items between groups
Zheng (2019)		Chinese	Parallel	BXT (40)	Domperidone (40)		1 month	① TCE	① 95% vs. 75% (*p* < 0.05)
								② TCM SS	② The BXT group was better than the control group in 14 items (*p* < 0.05)
Cai (2018)		Chinese	Parallel	BXT (43)	Trimebutine malate (43)		1 month	① TCE	① 88.37% vs. 60.77% (*p* < 0.05)
								② Symptom score	② total score 3.5 ± 1.2 vs. 5.2 ± 1.3 (*p* < 0.01) abdominal discomfort 1.3 ± 0.5 vs. 2.1 ± 0.6 postprandial fullness 1.5 ± 0.2 vs. 2.1 ± 0.1 anorexia 1.6 ± 0.1 vs. 2.2 ± 0.3
Deng (2016)		Chinese	Parallel	BXT (51)	Trimebutine malate (51)		not reported	① TCE	① 90.20% vs. 72.55% (*p* < 0.05)
Fu (2017)		Chinese	Parallel	BXT (54)	Trimebutine malate (40)		4 weeks	① TCE	① 92.59% vs. 75.00% (*p* < 0.05)
Li et al. (2013)		Chinese	Parallel	BXT (60)	Trimebutine malate (52)		4 weeks	① TCE	① 93.33% vs. 75.00% (*p* < 0.01)
Li (2016)		Chinese	Parallel	BXT (60)	Trimebutine malate (60)		4 weeks		① 90.00% vs. 75.00% (*p* < 0.05)
									② total score
								① TCE	9.7 ± 3.3 vs. 14.2 ± 4.1 (*p* < 0.05) postprandial fullness
								② Symptom score	2.3 ± 1.1 vs. 4.0 ± 11.4 (*p* < 0.05) early satiety
									2.1 ± 1.0 vs. 3.5 ± 1.2 (*p* < 0.05) epigastric pain
									1.5 ± 0.7 vs. 2.7 ± 0.9 (*p* < 0.05) epigastric burning
									2.4 ± 0.7 vs. 3.2 ± 1.0 (*p* < 0.05)
								③ AE	③ No serious adverse effects
Luo (2016)		Chinese	Parallel	BXT (40)	Trimebutine malate (40)		1 month	① TCE	① 97.5% vs. 80% (*p* < 0.05)
								② Index score	② The BXT group was better than the control group (*p* < 0.05)
Cai (2016)		Chinese	Parallel	BXT (30)	Mosapride (30)		4 weeks	① TCE	① 93.33% vs. 86.67% (*p* < 0.05)
								② TCM SS	② 5.67 ± 2.82 vs. 10.17 ± 3.06 (*p* < 0.05)
								③ Symptom score (main symptoms)	③ The BXT group was better than the control group (*p* < 0.05)
								④ Symptom score (secondary symptoms)	④ The BXT group was better than the control group (*p* < 0.05)
								⑤ HAMD	⑤ 5.37 ± 1.13 vs. 7.37 ± 1.63 (*p* < 0.05)
								⑥ AE	⑥ No adverse effects
Chen (2010)		Chinese	Parallel	BXT (56)	Mosapride (30)		1 month	① TCE	① 96.43% vs. 88.67% (*p* < 0.05)
Li and An (2016)		Chinese	Parallel	BXT (44)	Mosapride (40)		4 weeks	① TCE	① 95.5% vs. 77.5% (*p* < 0.05)
								② AE	② No difference between groups
Min (2009)		Chinese	Parallel	BXT (30)	Mosapride (30)		4 weeks	① TCE	① 90% vs. 73.3% (*p* < 0.05)
								② Symptom score	② 8.32 ± 3.14 vs.15.21 ± 4.13 (*p* < 0.01)
Nong (2017)		Chinese	Parallel	BXT (73)	Mosapride (73)		4 weeks	① TCE	① 93.15% vs. 73.97% *(p* < 0.05)
								② Symptom score	② belching
									1.31 ± 0.14 vs. 1.52 ± 0.25 (*p* < 0.05) bloating
									0.61 ± 0.16 vs. 1.53 ± 0.37 (*p* < 0.05) epigastric fullness
									1.52 ± 0.39 vs. 3.53 ± 0.71 *(p* < 0.05) epigastric pain
									0.83 ± 0.15 vs. 2.43 ± 0.47 (*p* < 0.05) epigastric burning
									0.72 ± 0.22 vs. 1.78 ± 0.52 *(p* < 0.05)
								③ MTL	③ 397.66 ± 54.280 vs. 283.13 ± 50.173 (*p* < 0.05)
Tang (2015)		Chinese	Parallel	BXT (37)	Mosapride (37)		6 weeks	① TCE	① 94.59% vs. 75.68% (*p* < 0.05)
								② AE	② No difference between groups
								③ R6MAT	③ 20.00% vs. 42.86% (*p* < 0.05)
Wang and Zhu (2007)		Chinese	Parallel	BXT (80)	Mosapride (80)		4 weeks	① TCE	① 93.75% vs. 78.75% (*p* < 0.01)
Wang et al. (2012)		Chinese	Parallel	BXT (43)	Mosapride (43)		2 weeks	① TCE	① 88.4% vs. 83.7% (*p* < 0.05)
Wang (2018)		Chinese	Parallel	BXT (35)	Mosapride (35)		30 days	① TCE	① 94.29% vs. 74.29% (*p* < 0.05)
								② Symptom score	② epigastric pain
									1.13 ± 0.24 vs. 1.82 ± 0.26 (*p* < 0.05) epigastric fullness
									1.12 ± 0.24 vs. 1.77 ± 0.34 (*p* < 0.05) acid reflux
									1.33 ± 0.32 vs. 2.16 ± 0.39 (*p* < 0.05)
								③ AE	③ 2.86% vs. 22.86% (*p* < 0.05)
Yi (2015)		Chinese	Parallel	BXT (28)	Mosapride (28)		30 days	① TCE (TCM symptoms aspect)	① 92.86% vs. 85.71% (*p* < 0.05)
								② TCE (overall)	② 82.14% vs. 71.43% (*p* < 0.05)
								③ TCM SS	③ 7.56 ± 2.35 vs. 11.69 ± 2.81 (*p* < 0.05)
								④ TCM SS (main symptoms)	④ The BXT group was better than the control group in 2 items (*p* < 0.05), and there was no difference in 1 item between groups
								⑤ TCM SS (secondary symptoms)	⑤ The BXT group was better than the control group in 4 items (*p* < 0.05), and there was no difference in 2 items between groups
								⑥ R3MAT	⑥ 10.71% vs. 28.57% (*p* = 0.005)
Zhang (2017)		Chinese	Parallel	BXT (40)	Mosapride (40)		28 days	① TCE	① 92.50% vs. 72.50% (*p* < 0.05)
								② TCM SS	② belching
									1.10 ± 0.25 vs. 1.78 ± 0.30 (*p* < 0.01) acid reflux
									1.14 ± 0.22 vs. 1.71 ± 0.16 (*p* < 0.01) epigastric pain
									1.28 ± 0.30 vs. 2.04 ± 0.31 (*p* < 0.01) epigastric fullness
									1.34 ± 0.41 vs. 2.24 ± 0.31 (*p* < 0.01)
Zhu and Gu (2008)		Chinese	Parallel	BXT (60)	Mosapride (60)		4 weeks	① TCE	① 93.33% vs. 75% (*p* < 0.05)
								② Symptom score	② The BXT group was better than the control group in 4 items (*p* < 0.05), and there was no difference in 2 items between groups
								③ MTL	③ 292.23 ± 47.87 vs. 271.07 ± 48.36 (*p* < 0.05)
Zou (2015)		Chinese	Parallel	BXT (64)	Mosapride (64)		4 weeks	① TCE	① 95.3% vs. 82.8% (*p* < 0.05)
								② Symptom score	② belching 1.1 ± 0.26 vs. 1.77 ± 0.31 (*p* < 0.05) acid reflux 1.13 ± 0.23 vs. 1.70 ± 0.17 (*p* < 0.05) epigastric fullness 1.33 ± 0.42 vs. 2.23 ± 0.32 (*p* < 0.05) epigastric pain 1.27 ± 0.31 vs. 2.03 ± 0.32 (*p* < 0.05)
Shi (2014)		Chinese	Parallel	BXT (35)	Cisapride (35)		4 weeks	① TCE	① 94.3% vs. 62.8% (*p* < 0.05)
Zhang (2010)		Chinese	Parallel	BXT (103)	Cisapride (95)		4 weeks	① TCE	① 97.1% vs. 90.5% (*p* < 0.05)
								② Symptom score	② The BXT group was better than the control group (*p* < 0.05)
Dong and Chen (2009)		Chinese	Parallel	BXT (63)	Domperidone Omeprazole on demand (46)		15 days	① TCE	① 93.65% vs. 78.26% (*p* < 0.05)
Fu (2010)		Chinese	Parallel	BXT (50)	Domperidone Omeprazole (50)		4 weeks	① TCE	① 100% vs. 90% (*p* < 0.01)
Lang and Cheng (2015)		Chinese	Parallel	CZBXT (49)	Domperidone Omeprazole (49)		14 days	① TCE	① 83.67% vs. 63.27% (*p* < 0.05)
								② Symptom score	② epigastric pain
									1.04 ± 0.26 vs. 1.79 ± 0.24 (*p* < 0.05) bloating
									0.70 ± 0.19 vs. 1.57 ± 0.26 (*p* < 0.05) belching
									0.85 ± 0.27 vs. 1.42 ± 0.35 (*p* < 0.05) poor oral intake
									0.98 ± 0.48 vs. 1.68 ± 0.49 (*p* < 0.05)
								③ 24 h pH monitoring	③ 2.31 ± 0.35 vs. 2.03 ± 0.23 (*p* < 0.05)
								④ AE	④ No serious adverse effects
Liang et al. (2010)		Chinese	Parallel	CZBXT (30)	Domperidone		2 weeks	① TCE	① 93.33% vs. 80.00% (*p* < 0.05)
					Omeprazole (30)			② 24 h pH monitoring	② No change in the CZBXT group, and pH elevated in the control group
Lu (2018)		Chinese	Parallel	BXT (50)	Domperidone Omeprazole (50)		4 weeks	① TCE	① 96% vs. 80% (*p* < 0.05)
								② TCM SS	② epigastric pain
									1.37 ± 0.31 vs. 1.69 ± 0.35 (*p* < 0.05) bloating
									1.55 ± 0.36 vs. 1.91 ± 0.38 (*p* < 0.05) belching
								③ MTL	1.34 ± 0.38 vs. 1.78 ± 0.41 (*p* < 0.05)
								④ Plasma gastrin	Poor oral intake
									1.36 ± 0.29 vs. 1.72 ± 0.37 (*p* < 0.05)
									③ 179.04 ± 45.27 vs. 132.45 ±40.31 (*p* < 0.05)
									④ 127.41 ± 29.86 vs. 94.92 ± 28.67 (*p* < 0.05)
Su (2014)		Chinese	Parallel	BXT (60)	Domperidone Lansoprazole (60)		4 weeks	① TCE	① 86.7% vs. 61.7% (*p* < 0.05)
								② Symptom score	② The BXT group was better than the control group (*p* < 0.05)
Yu (2016)		Chinese	Parallel	BXT (30)	Mosapride Rabeprazole (30)		4 weeks	① TCE	① 93. 3% vs. 70.00% (*p* < 0.05)
								② TCM SS	② 5.93 ± 4.78 vs. 8.93 ± 4.91(*p* < 0.01)
								③ TCM SS (main symptoms)	③ The BXT group was better than the control group in 2 items (*p* < 0.05), and there was no difference in 2 items between groups
								④ TCM SS (secondary symptoms)	④ The BXT group BXT was better than the control group in 2 items (*p* < 0.05), and there was no difference in 1 items between groups
								⑤ AE	⑤ No adverse effects
Huang and Long (2004)		Chinese	Parallel	BXT Domperidone (60)	Domperidone (60)		4 weeks	① TCE	① 93. 3% vs. 82.54% (*p* < 0.05)
								② Symptom score change	② 3.2 ± 3.6 vs. 2.4 ± 2.0 (*p* < 0.05)
Li et al. (2014)		Chinese	Parallel	BXT Domperidone (40)	Domperidone (40)		4 weeks	① TCE	① 90% vs. 80% (*p* < 0.05)
								② Symptom score	② abdominal pain
									0.72 ± 0.06 vs. 1.94 ± 0.81 (*p* < 0.05) bloating
									1.64 ± 0.08 vs. 3.17 ± 1.02 (*p* < 0.05) acid reflux
									0.62 ± 0.08 vs. 1.04 ± 0.33 (*p* < 0.05) belching
									0.95 ± 0.04 vs. 1.42 ± 0.32 (*p* < 0.05)
								③ MTL	③ 398.68 ± 120.14 vs. 304.23 ± 98.45 (*p* < 0.05)
								④ SCL-90 score	④ somatization
									18.82 ± 2.13 vs. 32.42 ± 4.22 (*p* < 0.05) obsessive-compulsive
									21.45 ± 2.92 vs. 32.92 ± 4.63 (*p* < 0.05) interpersonal sensitivity
									18.27 ± 2.59 vs. 29.64 ± 4.12 (*p* < 0.05) depression
									31.42 ± 4.52 vs. 44.51 ± 7.23 (*p* < 0.05) anxiety
									20.49 ± 3.61 vs. 31.14 ± 4.48 (*p* < 0.05) hostility
									21.51 ± 3.18 vs. 29.24 ± 3.61 (*p* < 0.05) phobic anxiety
									18.42 ± 2.52 vs. 27.21 ± 3.27 (*p* < 0.05) paranoid ideation
									16.14 ± 2.15 vs. 28.43 ± 3.44 (*p* < 0.05) psychoticism
									21.36 ± 3.22 vs. 34.42 ± 4.62 (*p* < 0.05)
Zhang et al. (2019)		Chinese	Parallel	BXT Domperidone (43)	Domperidone (43)		4 weeks	① TCE	① 95.35% vs. 76.74% (*p* < 0.05)
								② Symptom score	② abdominal pain
									0.78 ± 0.08 vs. 2.06 ± 0.87 (*p* < 0.05) bloating
									1.68 ± 0.42 vs. 3.21 ± 1.09 (*p* < 0.05) acid reflux
									0.78 ± 0.15 vs. 1.17 ± 0.26 (*p* < 0.05) belching
									0.93 ± 0.16 vs. 1.36 ± 0.47 (*p* < 0.05)
								③ MTL	③ 396.78 ± 118.69 vs. 312.46 ± 89.52 (*p* < 0.05)
								④ SCL-90 score	④ somatization
									18.82 ± 2.35 vs. 32.24 ± 4.10 (*p* < 0.05) obsessive-compulsive
									21.25 ± 2.87 vs. 31.25 ± 3.21 (*p* < 0.05) interpersonal sensitivity
									18.22 ± 3.15 vs. 29.01 ± 4.03 (*p* < 0.05) depression
									31.25 ± 3.16 vs. 44.21 ± 4.47 (*p* < 0.05) anxiety
									25.36 ± 5.21 vs. 43.02 ± 4.11 (*p* < 0.05) hostility
									21.08 ± 3.33 vs. 28.31 ± 3.17 (*p* < 0.05) phobic anxiety
									18.35 ± 4.87 vs. 27.66 ± 3.25 (*p* < 0.05) paranoid ideation
									16.25 ± 4.71 vs. 28.21 ± 4.53 (*p* < 0.05) psychoticism
									21.37 ± 5.16 vs. 34.51 ± 4.44 (*p* < 0.05)
Dang (2019)		Chinese	Parallel	BXT Mosapride (65)	Mosapride (65)		2 weeks	① TCE	① 93.9% vs. 80.0% (*p* < 0.05)
								② Symptom improvement time	② The experimental group was better than the control group (*p* < 0.05)
He and Xie (2012)		Chinese	Parallel	BXT Mosapride (62)	Mosapride (62)		4 weeks	① TCE	① 93.55% vs. 77.42% (*p* < 0.05)
								② Symptom score	② The experimental group was better than the control group in 5 items (*p* < 0.05), and there was no difference in 1 items between groups
								③ MTL	③ 391.26 ± 51.48 vs. 294.53 ± 52.63 (*p* < 0.05)
								④ AE	④ No serious adverse effects
Yin (2011)		Chinese	Parallel	BXT Mosapride (29)	Mosapride (29)		4 weeks	① TCE	① 93.10% vs. 72.41% (*p* < 0.05)
								② Symptom score	② 8.69 ± 4.89 vs. 9.52 ± 4.88 (*p* < 0.05)
								③ Symptom score (main symptoms)	③ The experimental group was better than the control group in 4 items (*p* < 0.05), and there was no difference in 2 items between groups
								④ TCM SS (secondary symptoms)	④ The experimental group was better than the control group in 2 items (*p* < 0.05), and there was no difference in 2 items between groups
Xie et al. (2011)		Chinese	Parallel	BXT Mosapride Omeprazole Amitriptyline on demand Clarithromycin on demand (42)	BXT Mosapride Omeprazole Amitriptyline on demand Clarithromycin on demand (40)		4 weeks	① TCE	① 95.2% vs. 77.5% (*p* < 0.05)
								② Symptom score	② The experimental group was better than the control group (*p* < 0.05)
								③ MTL	③ 399.52 ± 57.36 vs. 301.53 ± 65.23 (*p* < 0.01)
Kim (2017)		English	Parallel	BXT (25)	Placebo (23)		4 weeks	① NDI-K symptom score change	① −27.52 ± 22.11 vs. −22.83 ± 34.79 (*p* > 0.05)
								② VAS	② 34.92 ± 17.83 vs. 45.13 ± 12.22 (*p* > 0.05)
								③ NDI-K quality of life change	③ 8.02 ± 17.61 vs. 13.22 ± 13.38 (*p* > 0.05)
								④ FD-QOL	④ 66.81 ± 7.80 vs. 60.98 ± 12.04 (*p* > 0.05)
								⑤ EGG	⑤ No difference between groups
								⑥ AE	⑥ No difference between groups
Kim et al. (2021)		English	Parallel	BXT (15)	Placebo (16)		4 weeks	① NDI-K score improvement	① 37.40 ± 27.40 vs. 22.50 ± 23.85 (*p* = 0.12)
								② VAS (cm) change	② 3.19 ± 1.60 vs. 1.38 ± 2.85 (*p* = 0.03)
								③ Plasma ghrelin level change	③ 105.69 ± 287.89 vs. −142.31 ± 314.32 (*p* = 0.03)
								④ AE	④ No difference between groups
								⑤ VAS (mm)	⑤ 42.56 ± 16.35 vs. 54.4 ± 20.2 (*p* = 0.082)
Park et al. (2013)		English	Parallel	BXT (50)	Placebo (50)		6 weeks	① GIS score (total)	① 8.77 ± 6.87 vs. 6.83 ± 5.42 (*p* > 0.05)
								② GIS score (symptoms)	② The experimental group was better than the control group in 1 item (*p* < 0.05), and there was no difference in 9 items between groups
								③ VAS	③ 41.32 ± 18.21 vs. 34.54 ± 20.62 (*p* > 0.05)
								④ FD-QOL	④ 18.91 ± 17.58 vs. 18.51 ± 14.68 (*p* > 0.05)
								⑤ EGG	⑤ No difference between groups
								⑥ AE	⑥ No difference between groups
Dong (2018)		Chinese	Parallel	BXT Domperidone Flupentixol Melitracen (45)	Domperidone Flupentixol Melitracen (45)		4 weeks	① TCE	① 97.78% vs. 68.89% (*p* < 0.05)
								② QOL	② 98.56 ± 2.21 vs. 81.72 ± 2.53 (*p* < 0.05)
								③ Digestive function score	③ 8.19 ± 1.35 vs. 6.14 ± 1.12 (*p* < 0.05)
								④ Symptom disappearance time	④ The experimental group was better than the control group (*p* < 0.05)
								⑤ AE	⑤ No difference between groups
Huang (2011)		Chinese	Parallel	CZBXT (45)	Omeprazole (45)		2 weeks	① TCE	① 88.9% vs. 77.8% (*p* < 0.05)
								② AE	② The CZBXT group was better than the control group (*p* < 0.05)
Wang et al. (2019)		Chinese	Parallel	BXT (52)	Mosapride Estazolam (51)		4 weeks	① TCE	① 90.4% vs. 84.3% (*p* < 0.05)
								② Dyspepsia-related symptom score	② 4.58 ± 3.40 vs. 5.98 ± 3.39 (*p* < 0.05)
								③ PSQI	③ 4.60 ± 2.46 vs. 7.60 ± 3.18 (*p* < 0.05)
								④ AE	④ Only mild adverse effects in the BXT group, and 1 incomplete intestinal obstruction due to aggravation of constipation in the control group
Yu and Yu (2017)		Chinese	Parallel	BXT (100)	Trimebutine malate (100)		not reported	① Excellence rate	① 93% vs. 81% (*p* < 0.05)
								② AE	② 6% vs. 17% (*p* < 0.05)
Li and Li (2004)		Chinese	Parallel	BXT (30)		Cisapride (20)	30 days	① TCE	① 100% vs. 40% (*p* < 0.001)
Liang et al. (2008)		Chinese	Parallel	CZBXT (30)		Domperidone	2 weeks	① Symptom score (overall)	① 6.6000 ± 3.5389 vs. 11.633 ± 3.6717 (*p* < 0.01)
						Omeprazole (30)		② Symptom score (each symptoms)	② The CZBXT group was better than the control group in 3 items (*p* < 0.01), and there was no difference in 3 items between groups
Wang (2001)		Chinese	Parallel	BXT (60)		Domperidone Ranitidine (60)	4 weeks	① TCE	① 95.00% vs. 73.33% (*p* < 0.01)
Yang (2019)		Chinese	Parallel	BXT (25)		Octylonium Bromide (25)	4 weeds	① TCE	① 92.0% vs. 72.0% (*p* < 0.05)
									② epigastric pain
									1.24 ± 0.15 vs. 1.87 ± 0.19 (*p* < 0.05) belching
								② Symptom score	1.25 ± 0.21 vs. 1.68 ± 0.28 (*p* < 0.05) irritability
									1.47 ± 0.22 vs. 2.12 ± 0.25 (*p* < 0.05) bitter taste
									0.92 ± 0.32 vs. 1.68 ± 0.37 (*p* < 0.05)
Yang (2019)		Chinese	Parallel	BXT (48)		Compound Azintamide enteric-coated tablet (48)	1 month		① 95.83% vs. 68.75% (*p* < 0.05)
									② 3.91 ± 0.28 vs. 7.78 ± 0.62 (*p* < 0.05)
								① TCE	③ quality of sleep
								② Symptom score (overall)	69.45 ± 6.12 vs. 49.48 ± 6.09 (*p* < 0.05) mental state
								③ QOL	84.78 ± 8.62 vs. 63.91 ± 7.28 (*p* < 0.05) activity
									62.84 ± 5.83 vs. 51.48 ± 5.94 (*p* < 0.05) appetite
									70.54 ± 6.96 vs. 52.61 ± 7.02 (*p* < 0.05)
Tang (2014)		Chinese	Parallel	BXT Domperidone Omeprazole (29)		Domperidone Omeprazole (29)	4 weeks	① TCE	① 93.10% vs. 75.86% (*p* < 0.05)
								② Symptom score	② The experimental group was better than the control group (*p* < 0.05)
Ding (2018)		Chinese	Parallel	BXT Domperidone Flupentixol Melitracen (45)		Domperidone Flupentixol Melitracen (45)	4 weeks	① Satisfaction rate	① 95.56% vs. 80.00% (*p* < 0.05)
								② CRH	② 7.71 ± 0.32 vs. 10.14 ± 0.69 (*p* < 0.05)
								③ MTL	③ 172.21 ± 20.51 vs. 125.92 ± 20.25 (*p* < 0.05)
								④ TCM SS	④ 0.72 ± 0.11 vs. 2.11 ± 0.42 (*p* < 0.05)
Zhao et al. (2013)		English	Parallel	BXT (67)		Placebo (34)	4 weeks	① TDS	① gastroenterologist score
									2.37 ± 2.15 vs. 5.09 ± 3.00 (*p* < 0.01) patient score
									2.43 ± 1.98 vs. 5.13 ± 3.32 (*p* < 0.01)
								② SDS	② gastroenterologist score
									The BXT group was better than the placebo group (*p* < 0.05) patient score
									The BXT group was better than the placebo group (*p* < 0.05)
Tian (2017)		Chinses	Parallel	BXT (44)		Placebo (21)	4 weeks	① Water load test	① No difference between groups
								② Anxiety depression self-rating scale	② No difference between groups
								③ TCM SS	③ The BXT group was better than the control group in 3 items (*p* < 0.05), and there was no difference in 10 items between groups
								④ Symptom score	④ No difference between groups

BXT, *Banxia-xiexin tang*; TCE, Total clinical efficacy rate; MMC, Mucosal mast cell; MTL, Motilin; GE T1/2, Gastric emptying 1/2 time; SP, Substance P; CGRP, Calcitonin gene-related peptide; CZBXT, *Chaizhi-Banxia-xiexin tang*; TCM SS, Traditional Chinese medicine symptom scale; EGG, Electrogastrography; QOL, Quality of life; AE, Adverse effect; HAMD, Hamilton depression rating scale; R6MAT, Recurrence 6 months after treatment; SCL-90, Symptom checklist-90; NDI-K, Nepean dyspepsia index; VAS, Visual analog scale; FD-QOL: Functional dyspepsia-related quality of life; GIS, Gastrointestinal symptom; PSQI, Pittsburgh sleep quality index; CRH, Corticotropin-releasing hormone; TDS, Total dyspepsia symptom; SDS, Single dyspepsia symptom.

### 3.3 Assessment of risk of bias

**FIGURE 2:**
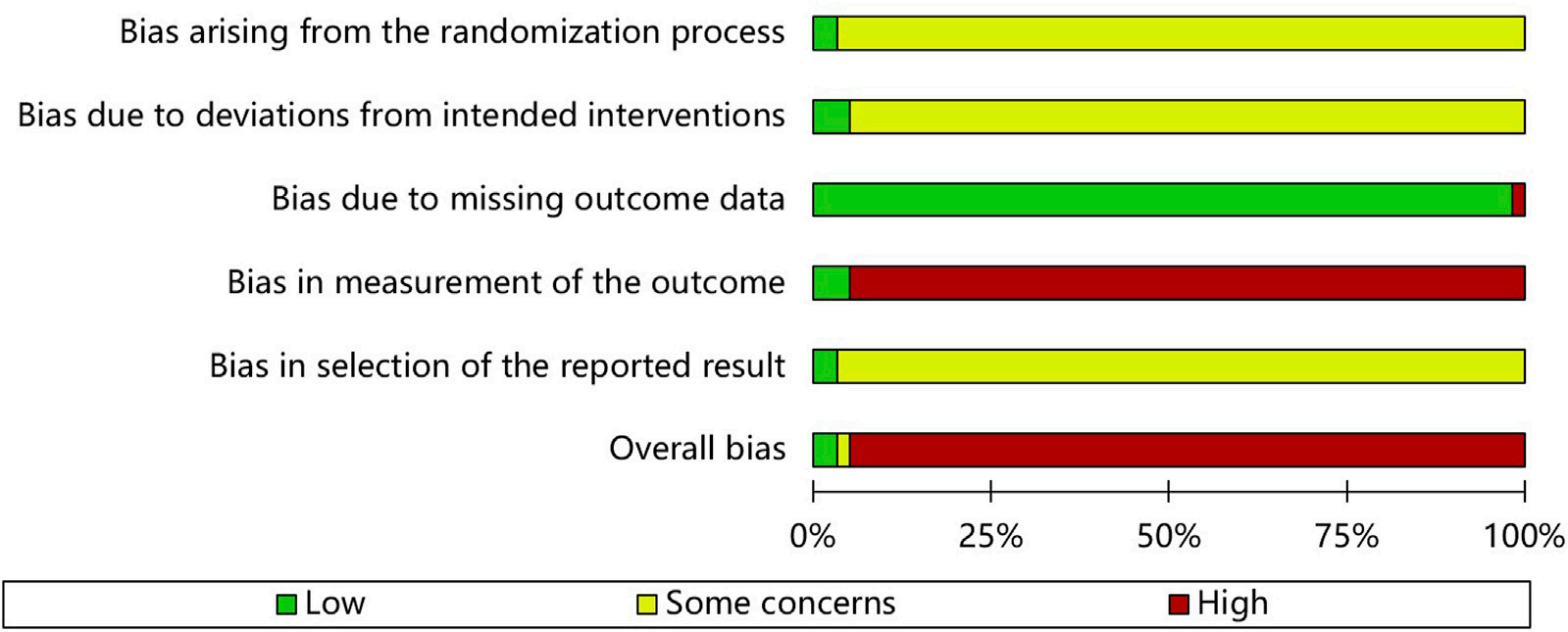
Risk of bias assessment graph.

Figure 2 shows the quality assessment results of the 57 studies selected for this meta-analysis using the RoB 2 tool. Table 2 shows the ratings for the individual domains at the study level for the 66 included studies.

**TABLE 2:** Risk of bias summary.

Study ID	Randomization process	Deviations from the Intended Interventions	Missing outcome data	Measurement of the outcome	Selection of the reported result	Overall
Dong *et al.* (2017)	Some concerns	Some concerns	Low risk	High risk	Some concerns	High risk
Feng *et al.* (2015)	Some concerns	Some concerns	Low risk	High risk	Some concerns	High risk
He (2007)	Some concerns	Some concerns	Low risk	High risk	Some concerns	High risk
Hu *et al.* (2006)	Some concerns	Some concerns	Low risk	High risk	Some concerns	High risk
Jin *et al.* (2004)	Some concerns	Some concerns	Low risk	High risk	Some concerns	High risk
Li (2015)	Some concerns	Some concerns	Low risk	High risk	Some concerns	High risk
Liu 2020	Some concerns	Some concerns	Low risk	High risk	Some concerns	High risk
Qiu (2011)	Some concerns	Some concerns	Low risk	High risk	Some concerns	High risk
Ren (2015)	Some concerns	Some concerns	Low risk	High risk	Some concerns	High risk
Tian (2018)	Some concerns	Some concerns	Low risk	High risk	Some concerns	High risk
Wu (2008)	Some concerns	Some concerns	Low risk	High risk	Some concerns	High risk
Yu and Yang (2010)	Some concerns	Some concerns	Low risk	High risk	Some concerns	High risk
Zhao and Song (2011)	Some concerns	Some concerns	Low risk	High risk	Some concerns	High risk
Zhao and Su (2017)	Some concerns	Some concerns	Low risk	High risk	Some concerns	High risk
Zheng (2019)	Some concerns	Some concerns	Low risk	High risk	Some concerns	High risk
Cai (2018)	Some concerns	Some concerns	Low risk	High risk	Some concerns	High risk
Deng (2016)	Some concerns	Some concerns	Low risk	High risk	Some concerns	High risk
Fu (2017)	Some concerns	Some concerns	Low risk	High risk	Some concerns	High risk
Li *et al.* (2013)	Some concerns	Some concerns	Low risk	High risk	Some concerns	High risk
Li (2016)	Some concerns	Some concerns	Low risk	High risk	Some concerns	High risk
Luo (2016)	Some concerns	Some concerns	Low risk	High risk	Some concerns	High risk
Cai (2016)	Some concerns	Some concerns	Low risk	High risk	Some concerns	High risk
Chen (2010)	Some concerns	Some concerns	Low risk	High risk	Some concerns	High risk
Li and An (2016)	Some concerns	Some concerns	Low risk	High risk	Some concerns	High risk
Min (2009)	Some concerns	Some concerns	Low risk	High risk	Some concerns	High risk
Nong (2017)	Some concerns	Some concerns	Low risk	High risk	Some concerns	High risk
Tang (2015)	Some concerns	Some concerns	Low risk	High risk	Some concerns	High risk
Wang and Zhu (2007)	Some concerns	Some concerns	Low risk	High risk	Some concerns	High risk
Wang *et al.* (2012)	Some concerns	Some concerns	Low risk	High risk	Some concerns	High risk
Wang (2018)	Some concerns	Some concerns	Low risk	High risk	Some concerns	High risk
Yi (2015)	Some concerns	Some concerns	Low risk	High risk	Some concerns	High risk
Zhang (2017)	Some concerns	Some concerns	Low risk	High risk	Some concerns	High risk
Zhu and Gu (2008)	Some concerns	Some concerns	Low risk	High risk	Some concerns	High risk
Zou (2015)	Some concerns	Some concerns	Low risk	High risk	Some concerns	High risk
Shi (2014)	Some concerns	Some concerns	Low risk	High risk	Some concerns	High risk
Zhang (2010)	Some concerns	Some concerns	Low risk	High risk	Some concerns	High risk
Dong and Chen (2009)	Some concerns	Some concerns	Low risk	High risk	Some concerns	High risk
Fu (2010)	Some concerns	Some concerns	Low risk	High risk	Some concerns	High risk
Lang and Cheng (2015)	Some concerns	Some concerns	Low risk	High risk	Some concerns	High risk
Liang *et al.* (2010)	Some concerns	Some concerns	Low risk	High risk	Some concerns	High risk
Lu (2018)	Some concerns	Some concerns	Low risk	High risk	Some concerns	High risk
Su (2014)	Some concerns	Some concerns	Low risk	High risk	Some concerns	High risk
Yu (2016)	Some concerns	Some concerns	Low risk	High risk	Some concerns	High risk
Huang and Long (2004)	Some concerns	Some concerns	Low risk	High risk	Some concerns	High risk
Li *et al.* (2014)	Some concerns	Some concerns	Low risk	High risk	Some concerns	High risk
Zhang *et al.* (2019)	Some concerns	Some concerns	Low risk	High risk	Some concerns	High risk
Dang (2019)	Some concerns	Some concerns	Low risk	High risk	Some concerns	High risk
He and Xie (2012)	Some concerns	Some concerns	Low risk	High risk	Some concerns	High risk
Yin (2011)	Some concerns	Some concerns	Low risk	High risk	Some concerns	High risk
Xie *et al.* (2011)	Some concerns	Some concerns	Low risk	High risk	Some concerns	High risk
Kim (2017)	Some concerns	Low risk	Low risk	Low risk	Some concerns	Some concerns
Kim *et al.* (2021)	Low risk	Low risk	Low risk	Low risk	Low risk	Low risk
Park *et al.* (2013)	Low risk	Low risk	Low risk	Low risk	Low risk	Low risk
Dong (2018)	Some concerns	Some concerns	Low risk	High risk	Some concerns	High risk
Huang (2011)	Some concerns	Some concerns	Low risk	High risk	Some concerns	High risk
Wang *et al.* (2019)	Some concerns	Some concerns	High risk	High risk	Some concerns	High risk
Yu and Yu (2017)	Some concerns	Some concerns	Low risk	High risk	Some concerns	High risk
Li and Li (2004)	Some concerns	Some concerns	Low risk	High risk	Some concerns	High risk
Liang *et al.* (2008)	Some concerns	Some concerns	Low risk	High risk	Some concerns	High risk
Wang (2001)	Some concerns	Some concerns	Low risk	High risk	Some concerns	High risk
Yang (2019)	Some concerns	Some concerns	Low risk	High risk	Some concerns	High risk
Yang (2019)	Some concerns	Some concerns	Low risk	High risk	Some concerns	High risk
Tang (2014)	Some concerns	Some concerns	Low risk	High risk	Some concerns	High risk
Ding (2018)	Some concerns	Some concerns	Low risk	High risk	Some concerns	High risk
Zhao *et al.* (2013)	Some concerns	Some concerns	Low risk	High risk	Some concerns	High risk
Tian (2017)	Some concerns	Low risk	Low risk	Some concerns	Some concerns	Some concerns

#### 3.3.1 Bias arising from the randomization process

Concerns were raised in 55 of the RCTs. These randomization studies did not report the sequence of allocation concealment. Two studies (Park et al., 2013; Kim et al., 2021) used a center-controlled method to randomize participants and were assessed to have a low RoB.

#### 3.3.2 Bias due to deviations from intended interventions

Three trials (Park et al., 2013; Kim, 2017; Kim et al., 2021) had low RoB because the participants and personnel were blinded to the placebo. Fifty-five studies had high RoB because participants and those delivering the interventions were aware of their assigned interventions. Deviations from the intended intervention occurred in these studies because of the trial context and group balance, which probably affected the outcome.

#### 3.3.3 Bias due to missing outcome data

**(** Wang et al., 2019) conducted a per-protocol analysis and had a high RoB. However, the other studies had a low RoB. Fifty-four trials did not have missing patients (Park et al., 2013). conducted an intention-to-treat analysis, while (Kim, 2017) had one missing patient before administering the test drug.

#### 3.3.4 Bias in measurement of the outcome

While three RCTs (Park et al., 2013; Kim, 2017; Kim et al., 2021) blinded outcome assessment, 54 did not report blinding of statistical analyses, and the outcome assessment was probably influenced by knowledge of the intervention received.

#### 3.3.5 Bias in selection of the reported result

Two RCTs (Park et al., 2013; Kim et al., 2021) with published study protocols had a low RoB. The remaining 55 studies were biased due to insufficient information.

#### 3.3.6 Overall bias

Of the 57 studies included in this meta-analysis, two had a low risk, one had a moderate risk, and 54 had a high overall RoB.

### 3.4 Primary outcome: total clinical efficacy rate

This systematic review examined the efficacy of BXT and combination therapy (BXT plus Western medicine) in treating FD using TCE.

#### 3.4.1 BXT *versus* western medicine

Western medicine groups were subdivided into PK agent groups, such as domperidone, trimebutine maleate, mosapride, and cisapride, as well as combinations of the PK and PPI groups. BXT was more efficacious than domperidone (RR: 1.19; 95% CI:1.11–1.21; *p* < 0.001), trimebutine maleate (RR: 1.24; 95% CI: 1.15–1.33; *p* < 0.001), mosapride (RR: 1.18; 95% CI: 1.13 to 1.24; *p* < 0.001), and combinations of PK and PPI (RR: 1.20; 95% CI: 1.12–1.29; *p* < 0.001). Heterogeneity was not significant in the trimebutine maleate (*p* = 0.99; *I* ^2^ = 0%), mosapride (*p* = 0.89; *I* ^2^ = 0%), or the combination of the PK and PPI groups (*p* = 0.31; *I* ^2^ = 16%).

**FIGURE 3:**
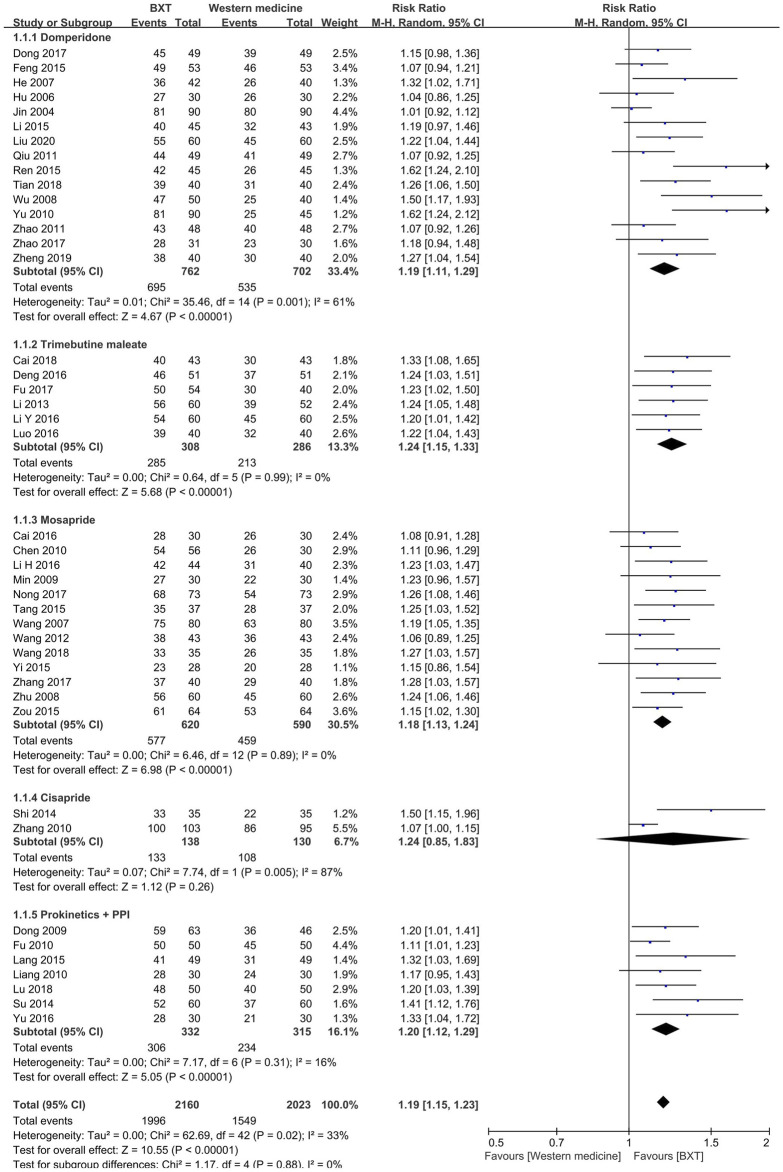
Forest plot of TCE between BXT and Western medicine. BXT: *Banxia-xiexin tang*; CI: confidence interval; TCE: total clinical efficacy rate.

In the integrated analysis of the five groups, 43 RCTs with 4,183 patients were included. They showed that BXT was more efficacious than Western medicine against FD (RR: 1.19; 95% CI: 1.15–1.23; *p* < 0.001) and had moderate heterogeneity (*p* = 0.02; *I* ^2^ = 33%) (Figure 3).

#### 3.4.2. Combination of BXT and western medicine *versus* western medicine

The Western medicine group was divided into two subgroups: domperidone and mosapride. The combination of BXT and Western medicine was more efficacious than domperidone (RR: 1.16; 95% CI: 1.06–1.27; *p* = 0.002) and mosapride (RR: 1.21; 95% CI: 1.10–1.33; *p* < 0.001). Heterogeneity was insignificant in either subgroup (*p* = 0.66, *I* ^2^ = 0%; *p* = 0.79, *I* ^2^ = 0%).

**FIGURE 4:**
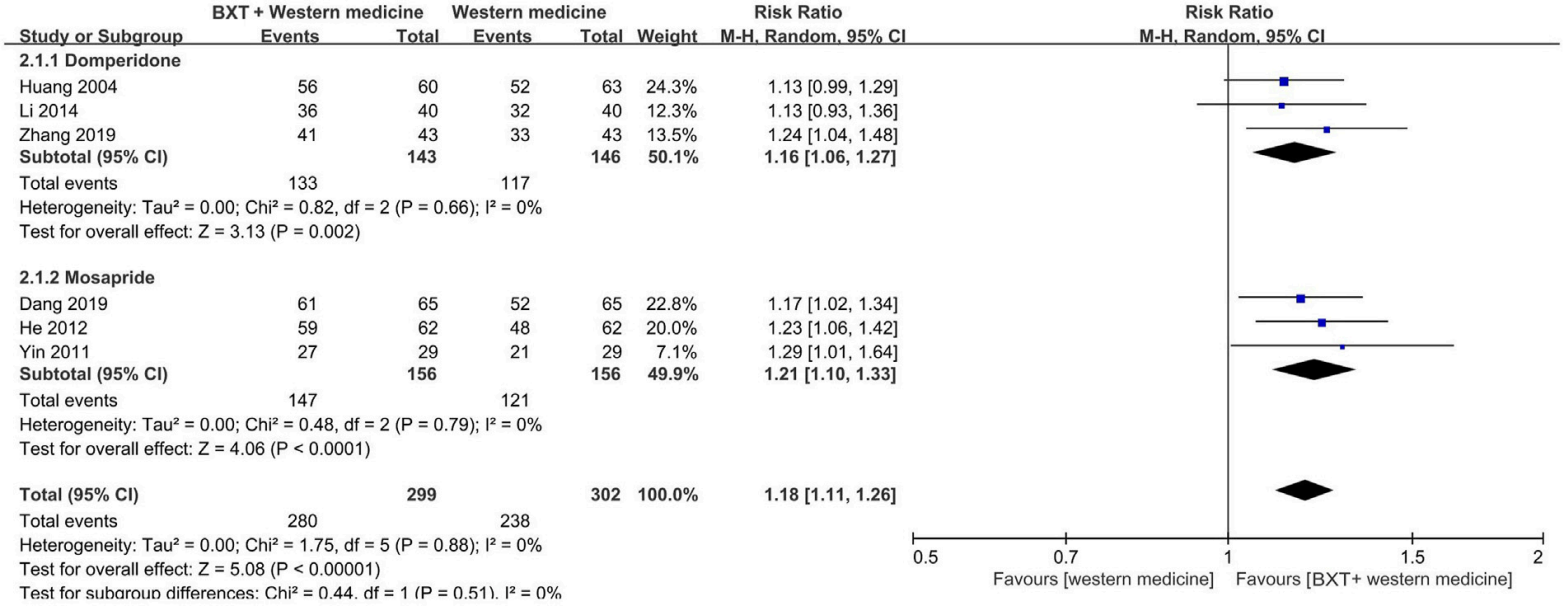
Forest plot of TCE between combination therapy and Western medicine alone. BXT: *Banxia-xiexin tang*; CI: confidence interval; TCE: total clinical efficacy rate.

Six studies with 601 patients were analyzed by integrating the subgroups. Combination therapy (BXT plus Western medicine) was more efficacious (RR: 1.18; 95% CI: 1.11–1.26; *p* < 0.001) and had lower heterogeneity (*p* = 0.88; *I* ^2^ = 0%) than Western medicine (Figure 4).

### 3.5 Motilin

**FIGURE 5:**
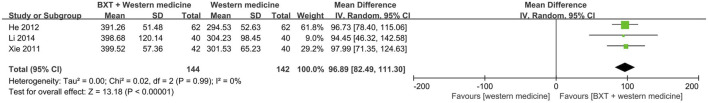
Forest plot of variation in motilin levels between combination therapy and Western medicine alone. BXT: *Banxia-xiexin tang*; SD: standard deviation; IV: inverse-variance; CI: confidence interval.

The meta-analysis included only plasma motilin levels (pg/ml) measured using radio-immunoassays before meals. Analysis of three RCTs with 286 participants showed that combination therapy (BXT plus Western medication) was more efficacious than Western medicine alone in boosting motilin secretion (MD: 96.89; 95% CI: 82.49–111.30; *p* < 0.001). Western medicine groups included domperidone (Li et al., 2014), mosapride (He and Xie, 2012), and a combination of mosapride, omeprazole, amitriptyline (on demand), and clarithromycin (on demand) (Xie et al., 2011). Heterogeneity was high (*p* < 0.001; *I* ^2^ = 0%) (Figure 5).

### 3.6 Symptom checklist-90-revised

Two studies (Li et al., 2014; Zhang et al., 2019) compared combination therapy (BXT plus domperidone) with domperidone alone and used the SCL-90-R to evaluate psychological symptoms. These studies included 166 patients. The SCL-90-R has nine subscales. Combination therapy (BXT plus domperidone) was more efficacious than domperidone alone in all aspects of the SCL-90-R.

**FIGURE 6:**
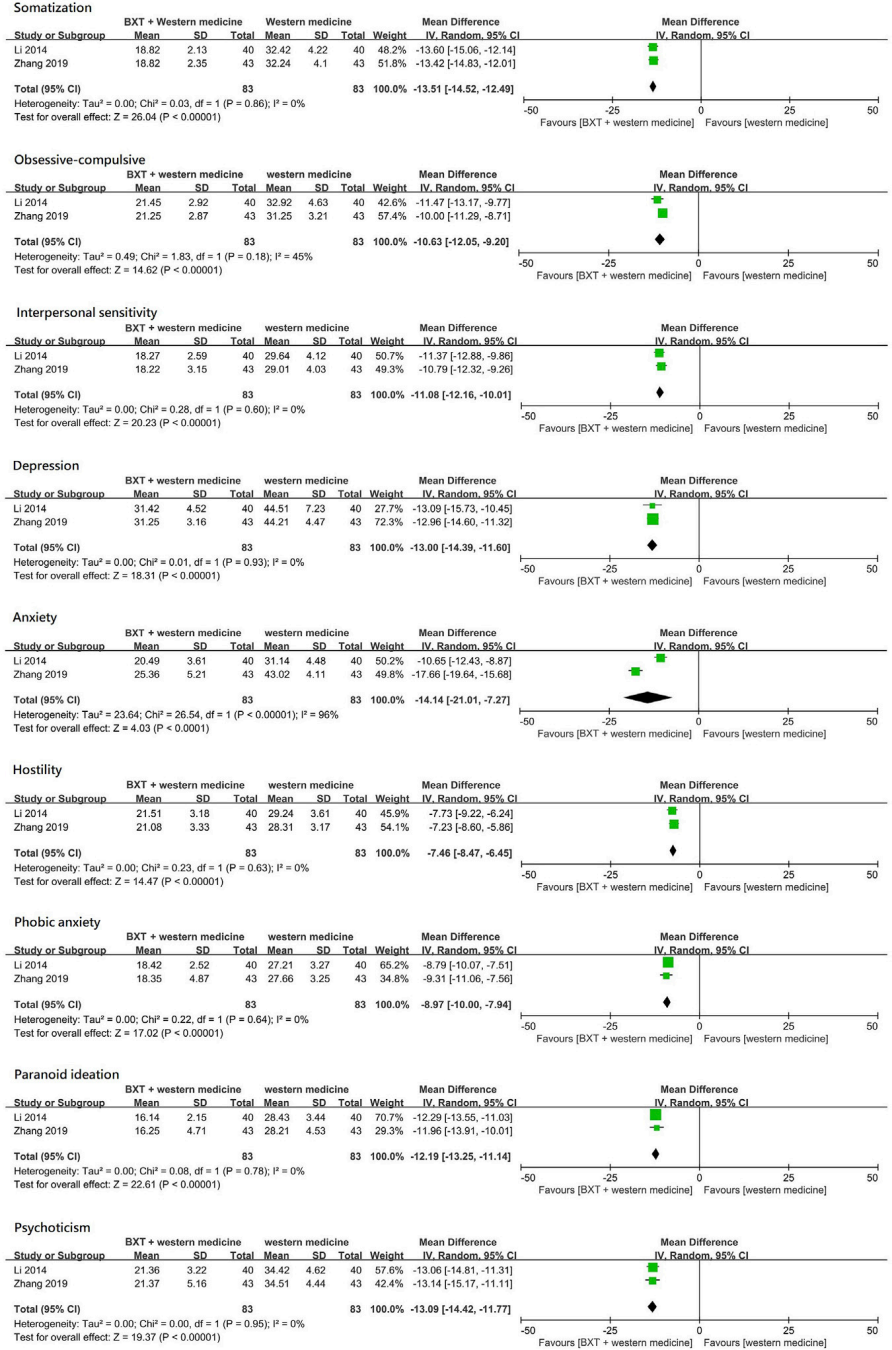
Forest plot of SCL-90-R between combination therapy and Western medicine alone. BXT: *Banxia-xiexin tang*; SD: standard deviation; IV: inverse-variance; CI: confidence interval.

The somatization score was significantly lower in the combination therapy group (BXT plus domperidone) than in the domperidone group (MD: –13.51; 95% CI: [–14.52]—[–12.49]; *p* < 0.001). Obsessive-compulsive symptoms improved remarkably in the combination therapy group (BXT plus domperidone) than in the domperidone group (MD: –10.63; 95% CI: [–12.05]- [–9.20]; *p* < 0.001), and interpersonal sensitivity was found to be more significantly improved in the combination therapy group than in the domperidone group (MD: –11.08; 95% CI: [–12.16] -[–10.01]; *p* < 0.001). Depression scores were significantly lower in the combination therapy group (BXT plus domperidone) than in the domperidone group (MD: –13.00; 95% CI: [–14.39]—[–11.60]; *p* < 0.001). Combination therapy (BXT plus domperidone) was found to be remarkably more efficacious against anxiety than domperidone (MD: –14.14; 95% CI: [–21.01]—[ −7.27]; *p* < 0.001) and reduced hostile symptoms more significantly than domperidone (MD: –7.46; 95% CI: [–8.47]—[–6.45]; *p* < 0.001). Combination therapy (BXT plus domperidone) was more efficacious in reducing phobic anxiety than domperidone (MD: –8.97; 95% CI [–10.00] - [–7.94]; *p* < 0.001). The paranoid ideation score was significantly lower in the combination therapy group (BXT plus domperidone) than in the domperidone group (MD: –12.19; 95% CI: [–13.25]—[–11.14]; *p* < 0.001). Combination therapy (BXT plus domperidone) was more efficacious in reducing the psychoticism score than domperidone (MD: –13.09; 95% CI [–14.42] - [–11.77]; *p* < 0.001) (Figure 6).

### 3.7 Visual analog scale

**FIGURE 7:**
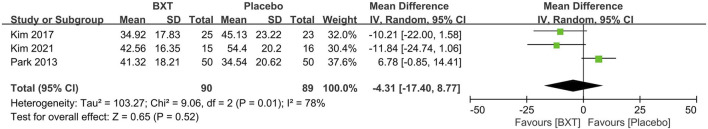
Forest plot of VAS between BXT and placebo. BXT: *Banxia-xiexin tang*; SD: standard deviation; IV: inverse-variance; CI: confidence interval.

Three RCTs (Park et al., 2013; Kim, 2017; Kim et al., 2021) with 179 participants compared the BXT and placebo groups using the VAS. No significant difference was observed between the BXT and placebo groups (MD: –4.31; 95% CI: [–17.40]—[–8.77]; *p* = 0.52). Heterogeneity was high (*p* = 0.01; *I* ^2^ = 78%) (Figure 7).

### 3.8 Subgroup analysis: *Chaizhi*-BXT *versus* western medicine

**FIGURE 8:**
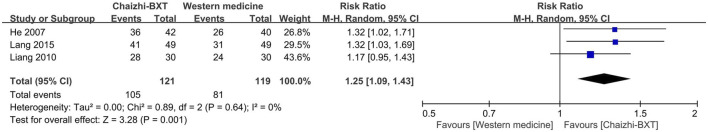
Forest plot of TCE between *Chaizhi*-BXT and Western medicine. BXT: *Banxia-xiexin tang*; CI: confidence interval; TCE: total clinical efficacy rate.

In this meta-analysis, the modified BXT group was integrated with the BXT group. A subgroup analysis was performed to minimize heterogeneity in the experimental groups. Three RCTs (He, 2007; Liang et al., 2010; Lang and Chen, 2015) with 240 participants showed that *Chaizhi*-BXT had a significantly higher TCE than Western medicine (RR: 1.25; 95% CI: 1.09–1.43; *p* = 0.001) (Figure 8).

### 3.9 Adverse events

**FIGURE 9:**
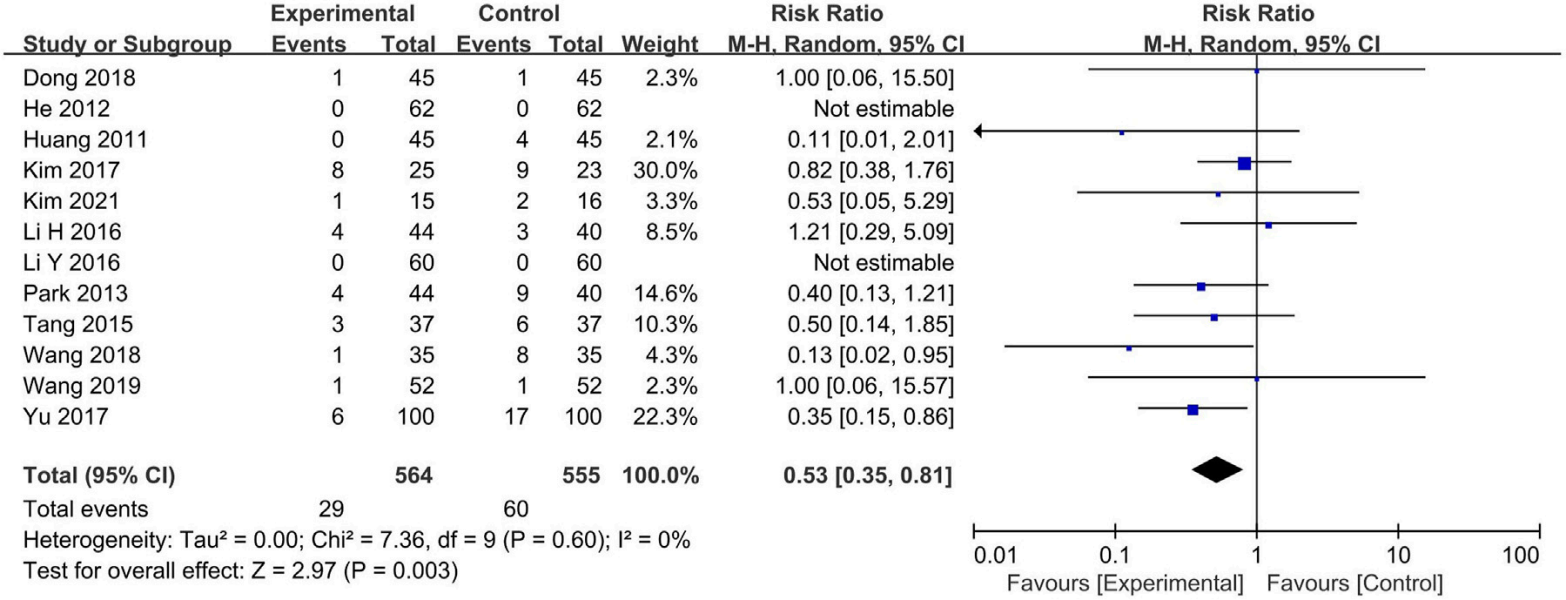
Adverse events. CI: confidence interval.

Of the 66 studies, 54 did not report any adverse events. The experimental group included BXT and Western medicine, and the control group included Western medicine and placebo. Two articles (He and Xie, 2012; Li, 2016) reported no adverse events, and one (Tang, 2015) reported the number of occurrences alone. The remaining nine RCTs reported mild adverse events. The number of adverse events was significantly lower in the experimental group than in the control group (RR: 0.53; 95% CI: 0.35–0.81; *p* = 0.003) (Figure 9).

Relatively mild adverse events such as nausea, vomiting, diarrhea, abdominal pain, acid reflux, epigastric fullness, dizziness, headache, urticaria, and insomnia were reported in the experimental groups. Adverse events in the control group were similar to those in the experimental groups and were mostly mild. However (Wang et al., 2019), reported intestinal obstruction (n = 1) due to progressive aggravation of constipation in the Western medicine (mosapride and estazolam) group.

### 3.10 Publication bias

**FIGURE 10:**
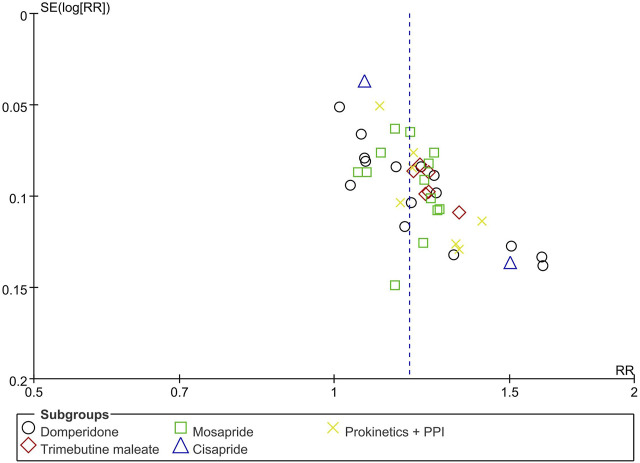
Funnel plot of TCE between BXT and Western medicine. BXT: *Banxia-xiexin tang*; RR: relative risk; TCE: total clinical efficacy rate.

Figure 10 shows a funnel plot of TCE comparing BXT and Western medicine. It is possible that smaller studies with lower estimates of benefits have not been published. However, the asymmetry is difficult to equate with publication bias because most of the included RCTs were published in Chinese, and the overall methodological quality of the studies was low.

### 3.11 Level of evidence

Table 3 shows the level of evidence for each outcome. Regarding TCE, BXT was more efficacious than Western medicine against FD. Because of the high RoB, the level of evidence was moderate. In the subgroup analysis, the level of evidence for BXT was lower than that of domperidone, considering the unexplained heterogeneity. The level of evidence for BXT was lower than that of cisapride due to unexplained heterogeneity and the small sample size. Combination therapy (BXT plus Western medicine) was more efficacious than Western medicine against FD. A high RoB led to a moderate level of evidence. In the subgroup analysis, the level of evidence was low because of the small sample size. Combination therapy (BXT plus Western medicine) was more efficacious than Western medicine alone in boosting motilin secretion. Because of the high RoB and the small sample size, the level of evidence was low. Combination therapy (BXT plus domperidone) had significantly better efficacy than domperidone in all aspects of the SCL-90-R. The level of evidence was low because of the high RoB and small sample size. The VAS scores showed no significant differences between the BXT and placebo groups. The RoB was low in the VAS; however, the small sample size led to a moderate level of evidence. The number of adverse events was significantly lower in the experimental group than in the control group. The level of evidence was moderate because of the high RoB.

**TABLE 3:** Level of evidence.

Variable		Certainty assessment					Effect		Certainty
		Number of studies	Risk of bias	Inconsistency	Indirectness	Imprecision	Relative (95% CI)	Absolute (95% CI)	
Total clinical efficacy	BXT vs. Western medicine	43	Serious	Not serious	Not serious	Not serious	RR 1.19 (1.15–1.23)	145 more per 1,000 (from 115 more to 176 more)	Moderate
	BXT vs. Domperidone	15	Serious	Serious	Not serious	Not serious	RR 1.19 (1.11–1.29)	145 more per 1,000 (from 84 more to 221 more)	Low
	BXT vs. Trimebutine maleate	6	Serious	Not serious	Not serious	Not serious	RR 1.24 (1.15–1.33)	179 more per 1,000 (from 112 more to 246 more)	Moderate
	BXT vs. Mosapride	13	Serious	Not serious	Not serious	Not serious	RR 1.18 (1.13–1.24)	140 more per 1,000 (from 101 more to 187 more)	Moderate
	BXT vs. Cisapride	2	Serious	Serious	Not serious	Serious	RR 1.24 (0.85–1.83)	199 more per 1,000 (from 125 fewer to 690 more)	Very low
	BXT vs. Prokinetics + PPI	7	Serious	Not serious	Not serious	Not serious	RR 1.20 (1.12–1.29)	149 more per 1,000 (from 89 more to 215 more)	Moderate
	CZBXT vs. Western medicine	3	Serious	Not serious	Not serious	Serious	RR 1.25 (1.09–1.43)	More per 1,000 (from 61 more to 1293 more)	Low
	BXT + Western medicine vs. Western medicine	6	Serious	Not serious	Not serious	Not serious	RR 1.18 (1.11–1.26)	142 more per 1,000 (from 87 more to 205 more)	Moderate
	BXT + Domperidone vs	3	Serious	Not serious	Not serious	Serious	RR 1.16 (1.06–1.27)	128 more per 1,000 (from 48 more to 216 more)	Low
	Domperidone								
	BXT + Mosapride vs	3	Serious	Not serious	Not serious	Serious	RR 1.21 (1.10–1.33)	163 more per 1,000 (from 78 more to 256 more)	Low
	Mosapride								
Motilin	BXT + Western medicine vs	3	Serious	Not serious	Not serious	Serious	-	MD 96.89 higher (82.49 higher to 111.3 higher)	Low
	Western medicine								
SCL-90-R	BXT + Western medicine vs								
	Western medicine								
Somatization		2	Serious	Not serious	Not serious	Serious	-	MD 13.51 lower (14.52 lower to 12.49 lower)	Low
Obsessive-compulsive		2	Serious	Not serious	Not serious	Serious	-	MD 10.63 lower (12.05 lower to 9.2 lower)	Low
Interpersonal sensitivity		2	Serious	Not serious	Not serious	Serious	-	11.08 lower (12.16 lower to 10.01 lower)	Low
Depression		2	Serious	Not serious	Not serious	Serious	-	MD 13 lower (14.39 lower to 11.6 lower)	Low
Anxiety		2	Serious	Not serious	Not serious	Serious	-	MD 14.14 lower (21.01 lower to 7.27 lower)	Low
Hostility		2	Serious	Not serious	Not serious	Serious	-	MD 7.46 lower (8.47 lower to 6.45 lower)	Low
Phobic anxiety		2	Serious	Not serious	Not serious	Serious	-	MD 8.97 lower (10 lower to 7.94 lower)	Low
Paranoid ideation		2	Serious	Not serious	Not serious	Serious	-	MD 12.19 lower (13.25 lower to 11.14 lower)	Low
Psychoticism		2	Serious	Not serious	Not serious	Serious	-	MD 13.09 lower (14.42 lower to 11.77 lower)	Low
VAS	BXT vs. placebo	3	Not serious	Not serious	Not serious	Serious	-	MD 4.31 lower (17.4 lower to 8.77 higher)	Moderate
Adverse effect	Experimental vs. control	12	Serious	Not serious	Not serious	Not serious	RR 0.53 (0.35–0.81)	51 fewer per 1,000 (from 70 fewer to 21 fewer)	Moderate

CI, confidence interval; BXT, *Banxia-xiexin tang*; RR, Relative risk; PPI, Proton pump inhibitor; CZBXT, *Chaizhi-Banxia-xiexin tang*; MD, Mean difference; SCL-90-R, Symptom checklist-90-revised.

## 4 Discussion

### 4.1 Summary of the main findings

This systematic review investigated the efficacy and safety of BXT and combination therapy (BXT plus Western medicine) against FD. The TCE results showed that BXT and combination therapy (BXT plus Western medicine) had a stronger therapeutic effect on FD than Western medicine alone. The motilin assays results showed that the combination therapy (BXT plus Western medicine) had more clinical benefits than Western medicine alone. Combination therapy (BXT plus domperidone) was more efficacious than domperidone alone in SCL-90-R. In the subgroup analysis, the *Chaizhi*-BXT group was more efficacious than the Western medicine group based on TCE. No significant difference was observed between the BXT and placebo groups regarding the VAS scores. None of the included RCTs reported severe adverse events in the BXT and combination therapy (BXT plus Western medicine) groups. The incidence of adverse reactions was lower in the BXT and combination therapy (BXT plus Western medicine) groups than in the Western medicine and placebo groups.

### 4.2 Comparison with previous reviews

Several previous studies have reported the efficacy and safety of BXT for treating FD. One meta-analysis, including 10 RCTs, reported that BXT was more efficacious than Western medicines (domperidone, mosapride, and a combination of domperidone and omeprazole) against FD. No adverse events were reported in the BXT group. However, side effects, such as GI symptoms and headaches, occurred in the control group (Gan et al., 2014). Another systematic review involving 30 studies from Chinese databases compared BXT with Western medicines, including PK agents (domperidone, mosapride, trimebutine, and cisapride), PPIs (omeprazole and lansoprazole), and H2 receptor antagonists (famotidine). The review reported that BXT was better than Western medicine in terms of TCE, recovery rate, and symptom improvement. However, there were no significant differences between the groups when plasma motilin levels and gastric dynamics (Li and Li, 2016) were considered. A systematic review of 20 trials indicated that Chinese herbal medicines, including modified BXT, were more efficacious than conventional Western medicines against FD and did not cause side effects (Zhang, 2015). A systematic review of 28 RCTs reported that BXT was more efficacious than Western medicine in increasing the TCE, reducing several symptoms and recurrence rates. However, BXT was less efficacious than Western medicine in reducing symptoms, such as early satiation and plasma motilin levels (Hu et al., 2020).

### 4.3 Components of BXT

BXT is a complex of seven botanical drugs: Pinellia ternata (Thunb.) Makino [Araceae; Pinellia ternata rhizoma], Panax ginseng C.A.Mey. [Araliaceae; Panax ginseng root], Zingiber officinale Roscoe [Zingiberaceae; Zingiber officinale rhizoma], Coptis chinensis Franch. [Ranunculaceae; Coptis chinensis rhizoma], Scutellaria baicalensis Georgi [Lamiaceae; Scutellaria baicalensis root], Glycyrrhiza uralensis Fisch. ex DC. [Fabaceae; Glycyrrhiza uralensis root], Ziziphus jujuba Mill. [Rhamnaceae; Zizyphus jujuba fruit] (Park et al., 2010). The pharmacological effects of Pinellia ternata (Thunb.) Makino [Araceae; Pinellia ternata rhizoma], such as anti-vomiting, anti-coughing, antidepressant, anti-inflammatory, and sedative-hypnotic activities, have been demonstrated in modern pharmacological studies (Mao and He, 2020). Scutellaria baicalensis Georgi [Lamiaceae; Scutellaria baicalensis root] contains several flavones, including baicalin, baicalein, wogonin, and wogonoside, and is known to have anti-inflammatory, anti-tumor, and anticonvulsant effects (Li and Zuo, 2011). Zingiber officinale Roscoe [Zingiberaceae; Zingiber officinale rhizoma], the root of ginger, has anti-emetic, anti-diarrheal, anti-oxidative, anti-inflammatory, anti-tumor, and anti-lipidemic effects (Chrubasik et al., 2005). The therapeutic effects of BXT against FD may be attributed to the efficacy of each BXT component. Combinations of various active ingredients may have advantages over other treatment options against FD, considering its heterogeneous pathophysiology and symptoms (Rösch et al., 2006). Additionally, interactions between multiple bioactive components in the herbal medicine formula can create synergistic effects, and further studies are required to reveal these interactions (Zhou et al., 2016).

### 4.4 Implication for the clinical practice

The pathophysiology of FD is unknown; however, the deterioration of gastric motility is considered one of the leading causes of FD symptoms (Vanheel and Farré, 2013). GI hormones, such as gastrin, somatostatin, and motilin, regulate secretion, digestion, absorption, appetite, and GI motility (Ahmed and Ahmed, 2019). Motilin stimulates antral contractions and improves gastric emptying (Van den Houte et al., 2020). This systematic review showed that BXT increases motilin secretion. In a previous pharmacological study, BXT enhanced somatostatin- and motilin-immunoreactive substances levels in human plasma (Naito et al., 2002). These results suggest that BXT may affect FD by normalizing gastric motility. However, further studies using sensitive measurements of gastric emptying, such as scintigraphy, isotope respiration tests, ultrasonography, and magnetic resonance imaging, are required to investigate the association between BXT and gastric motility.

The brain-gut axis plays an essential role in the pathophysiology of FD (Wauters et al., 2020). FD is associated with central modulation (brain-to-gut) and visceral sensory signaling (gut-to-brain). Bidirectional pathways are likely regulated by psychological influences and stress responses of the hypothalamic-pituitary-adrenal (HPA) axis (Van Oudenhove and Aziz, 2013; Wauters et al., 2020). Several epidemiological studies have reported a higher prevalence of psychiatric disorders in patients with FD than in healthy individuals (Van Oudenhove and Aziz, 2013). In some patients, mood or anxiety disorders precede the occurrence of FGID (Jones et al., 2017), and FD symptoms can induce anxiety and depression. A previous prospective study reported that anxiety was an independent predictor of FGID (Koloski et al., 2012).

The SCL-90-R is a self-rating clinical symptom scale of 90 questions associated with nine psychiatric subsections. In a previous study, the SCL-90-R was used to evaluate psychiatric distress in patients with FD, and the FD group had significantly higher scores than the healthy control group in all subsections (Faramarzi et al., 2014). In this systematic review, combination therapy (BXT plus domperidone) was better than domperidone alone in improving the SCL-90-R scores in patients with FD. The therapeutic effects of BXT on dyspeptic symptoms may relieve stress in patients and decrease their SCL-90-R scores. However, BXT exerts regulatory effects on the HPA axis by regulating the plasma cortisol and adrenocorticotropic hormone levels under stressful conditions (Naito et al., 2003).

Furthermore, a recent network pharmacological study reported that BXT could influence the process of depression by modulating the 5-hydroxytryptamine synaptic signaling pathway, arachidonic acid metabolism, and hypoxia-inducible factor-1 signaling pathway (Yu et al., 2020). One RCT suggested that combination therapy with BXT and antidepressants (paroxetine) had a more rapid effect than antidepressants (paroxetine) alone in female patients with somatoform disorders (Sun and Li, 2014). In another trial, combination therapy (BXT, flupentixol, and melitracen) was found to be more efficacious than Western medicines (flupentixol and melitracen) in reducing anxiety and depression, as well as improving the quality of life of perimenopausal depressed patients (Chen, 2019). BXT improved the psychological state of the patients and provided symptomatic relief. However, human data supporting the brain-gut axis are limited (Wauters et al., 2020). Further studies are required to investigate the interaction between the gut and brain in humans and the effects of BXT on bidirectional pathways.

### 4.5 Strengths of this study

This study has several strengths. First, it included the latest results on the effects of BXT against FD. Although a previous meta-analysis published in 2016 used Chinese databases alone (Li and Li, 2016), we searched global databases and did not apply language restrictions. Second, the superior effect of combination therapy (BXT plus Western medicine) against FD was indicated in this systematic review. Previous studies have compared the effect of BXT *versus* Western medicine or placebo; however, this analysis examined the synergistic effect of BXT and Western medicine on FD. Conventional medications are less efficacious in managing the symptoms of FD, and the demand for complementary medicine, such as herbal medicine, is increasing (Suzuki et al., 2009). In some patients, herbal and conventional Western medicines are being co-administered. Therefore, it is important to investigate the efficacy and safety of combination therapy (herbal medicine plus conventional Western medicine) for treating FD. Third, a subgroup analysis was performed for both the BXT and control groups. *Chaizhi*-BXT was found to be more efficacious than Western medicine. The Western medicine group was further subcategorized into domperidone, trimebutine maleate, mosapride, cisapride, and a combination of PK and PPI groups. Although cisapride was withdrawn from the global market because of its severe side effects on the cardiovascular system, it was included in the subgroup analysis to focus on the therapeutic effects of BXT against FD. Fourth, this review suggests that BXT could treat FD in addition to conventional Western medicine.

The American College of Gastroenterology and Canadian Association of Gastroenterology guidelines on dyspepsia recommend *H. pylori* eradication, if positive, as the first line of treatment. As secondary strategies, PPI, tricyclic antidepressants, and PK agents have been recommended for treating FD (Moayyedi et al., 2017). The Korean clinical guidelines strongly recommend PK agents and PPIs for treating FD. As a first-line treatment, PK agents for patients with postprandial distress syndrome (PDS) and PPI for patients with epigastric pain syndrome (EPS) have been suggested (Oh et al., 2020). PK agents constitute a significant part of the Western medicine used in this study. BXT and combination therapy (BXT plus Western medicine) were more efficacious than Western medicine alone. Furthermore, the psychological effects of BXT may benefit patients with FD.

### 4.6 Limitations and implications for further research

This systematic review has some limitations. First, the general methodological quality of the included RCTs was low because of moderate or high RoB in the randomization process, deviations from intended interventions, and measurement of the outcome domains. Most studies did not have a pre-published study protocol or sufficient information to assess the risk of reporting bias. Double-blinding was performed in five of the 66 studies, which resulted in poor methodological quality. Second, all the included studies were published in Asia, and most of them were from China. Third, clinical heterogeneity may exist among the intervention groups. Modified BXTs, which varied by species and the number of added botanical drugs, were included in the meta-analysis. High heterogeneity was observed in VAS scores among the outcomes. It is difficult to resolve the heterogeneity because of the small sample size; therefore, it is necessary to include more participants in future studies. In addition, the effects of different doses of BXT could not be compared due to the heterogeneity of constituent herbs and their doses. Fourth, in the included RCTs, there is lack of description of extraction procedure and quality control of the botanical drugs. Finally, pattern identification was not considered in the meta-analysis. In traditional Chinese medicine, FD can be differentiated into several patterns based on clinical signs and symptoms. BXT has often been regarded as an herbal formula treating “Mixed cold and heat” FD patterns (Ji et al., 2017). However, it might have different effects on other FD patterns. Consequently, well-designed RCTs with clear randomization and double-blinding are required.

## 5 Conclusion

BXT and combination therapy (BXT plus Western medicines) may have therapeutic effects against FD. BXT can be considered a treatment option for FD with fewer adverse events. However, the methodological quality of the included studies was low; hence, the validity of the evidence obtained is controversial. More robust, large-scale, high-quality RCTs are required for more credible evidence.

## Data Availability

The original contributions presented in the study are included in the article/Supplementary Material, further inquiries can be directed to the corresponding author.
