# Supplementary material for: Herbal medicine, Banxia-xiexin tang, for functional dyspepsia: a systematic review and meta-analysis
Source: Front Pharmacol. 2023 May 19;14:1130257. doi: 10.3389/fphar.2023.1130257 (PMC10235465; doi:10.3389/fphar.2023.1130257)
Supplement: Supplementary file 1 [file DataSheet1.DOCX]

**Supplementary material.** Information on *Banxia-xiexin tang* in included studies

| **Study ID** | | **Formulation** | **Extraction** | **Daily Dose of Botanical Drugs** | **Number of Doses per Day** | **Daily Dose of Extract** | |
| --- | --- | --- | --- | --- | --- | --- | --- |
| Dong et al*.*, 2017 | | Decoction | Water Boil the daily dose in 400ml of water | Pinelliae tuber 15g, Ginseng radix 9g, Zingiberis rhizoma 9g, Scutellariae radix 9g, Coptidis rhizome 3g, Glycyrrhizae radix 9g, 4 Zizyphi fructus | 2 | Not reported | |
| Feng et al*.*, 2015 | | Decoction | Water | Pinelliae tuber 15g, Ginseng radix 9g, Zingiberis rhizoma 9g, Scutellariae radix 9g, Coptidis rhizome 3g, Glycyrrhizae radix 9g, 4 Zizyphi fructus | 2 | 300ml | |
| He, 2007 | | Decoction | Water | Pinelliae tuber 10g, Codonopsis Pilosulae radix 15g, Zingiberis rhizoma 10g, Coptidis rhizome 6g, Glycyrrhizae radix 6g, Zizyphi fructus 5g, Bupleuri radix 10g, Ponciri fructus 10g, Atractylodis rhizoma alba 10g | 2 | 200ml | |
| Hu et al*.*, 2006 | | Decoction | Water | Pinelliae tuber 12g, Codonopsis Pilosulae radix 10g, Zingiberis rhizoma 5g, Scutellariae radix 5g, Coptidis rhizome 10g, 5 Zizyphi fructus, Magnoliae cortex 10g, Amomi fructus 10g, Paeoniae radix 10g, Citri Unshius pericarpium 10g, Atractylodis rhizoma alba 10g | 3 | 300ml | |
| Jin et al*.*, 2004 | | Granules | Not reported Granules were prepared by preparation room of Heilongjiang Academy of Traditional Chinese Medicine | Pinelliae tuber, Ginseng radix, Zingiberis rhizoma, Scutellariae radix, Coptidis rhizome, Glycyrrhizae radix, Zizyphi fructus  Dosages by botanical drugs are not reported. Dosage of one pack of granules is 10g | 3 | 30g | |
| Li, 2015 | | Decoction | Water Soak a daily dose in cold water for 30 minutes and then boil. | Pinelliae tuber 10g, Codonopsis Pilosulae radix15g, Zingiberis rhizoma 10g, Scutellariae radix 10g, Coptidis rhizome 6g, Glycyrrhizae radix 6g, Zizyphi fructus 10g | 3 | 300ml | |
| Liu 2020 | Decoction | | Water | Pinelliae tuber 12g, Codonopsis Pilosulae radix 15g, Zingiberis rhizoma 10g, Scutellariae radix 10g, Coptidis rhizome 5g, Glycyrrhizae radix 10g, 4 Zizyphi fructus | 2 | 300ml | |
| Qiu, 2011 | Decoction | | Water | Pinelliae tuber 12g, Codonopsis Pilosulae radix 10g, Scutellariae radix 10g, Coptidis rhizome 5g, Glycyrrhizae radix 10g, 4 Zizyphi fructus | 2 | 200ml | |
| Ren, 2015 | Decoction | | Water | Pinelliae tuber 15g, Ginseng radix 6g, Zingiberis rhizoma 5g, Scutellariae radix 10g, Coptidis rhizome 3g, Glycyrrhizae radix 9g, Zizyphi fructus 5g | 2 | Not reported | |
| Tian, 2018 | Decoction | | Water | Pinelliae tuber 10g, Codonopsis Pilosulae radix 6g, Zingiberis rhizoma 8g, Scutellariae radix 8g, Coptidis rhizome 12g, Glycyrrhizae radix 12g, Zizyphi fructus (not reported) | 2 | 300ml | |
| Wu, 2008 | | Decoction | Water | Pinelliae tuber 12g, Codonopsis Pilosulae radix 9g, Zingiberis rhizoma 6g, Scutellariae radix 9g, Coptidis rhizome 6g, Glycyrrhizae radix 6g, 4 Zizyphi fructus | 3 | 300ml | |
| Yu and Yang,  2010 | | Decoction | Water | Pinelliae tuber 15g, Codonopsis Pilosulae radix 20g, Zingiberis rhizoma 10g, Scutellariae radix 10g, Coptidis rhizome 5g, Glycyrrhizae radix 10g, 4 Zizyphi fructus | 2 | Not reported | |
| Zhao and Song,  2011 | | Decoction | Water | Pinelliae tuber 15g, Codonopsis Pilosulae radix 20g, Zingiberis rhizoma 10g, Scutellariae radix 10g, Coptidis rhizome 5g, Glycyrrhizae radix 10g, 4 Zizyphi fructus | 2 | 200ml | |
| Zhao and Su,  2017 | | Decoction | Water | Pinelliae tuber 12g, Codonopsis Pilosulae radix 15g, Zingiberis rhizoma 10g, Scutellariae radix 12g, Coptidis rhizome 6g, Glycyrrhizae radix 8g, Zizyphi fructus 3g | 2 | 400ml | |
| Zheng, 2019 | | Decoction | Water | Pinelliae tuber 15g, Codonopsis Pilosulae radix 10g, Zingiberis rhizoma 10g, Scutellariae radix 10g, Coptidis rhizome 4g, Glycyrrhizae radix 10g, 5 Zizyphi fructus | 2 | 300ml | |
| Cai, 2018 | | Decoction | Water | Pinelliae tuber 12g, Ginseng radix 9g, Zingiberis rhizoma 9g, Coptidis rhizome 8g, Glycyrrhizae radix 10g, 12 Zizyphi fructus, Astragali radix 9g | 2 | 300ml | |
| Deng, 2016 | | Decoction | Water | Pinelliae tuber 9g, Codonopsis Pilosulae radix 12g, Zingiberis rhizoma 10g, Scutellariae radix 12g, Coptidis rhizome 12g, Glycyrrhizae radix 5g, 4 Zizyphi fructus | 2 | Not reported | |
| Fu, 2017 | | Decoction | Water | Pinelliae tuber 10g, Codonopsis Pilosulae radix 12g, Zingiberis rhizoma 10g, Scutellariae radix 10g, Coptidis rhizome 8g, Glycyrrhizae radix 6g, Zizyphi fructus 12g | 2 | 300ml | |
| Li et al*.*, 2013 | | Decoction | Water | Pinelliae tuber 10g, Codonopsis Pilosulae radix 12g, Zingiberis rhizoma 10g, Scutellariae radix 10g, Coptidis rhizome 8g, Glycyrrhizae radix 6g, Zizyphi fructus 12g | 2 | 300ml | |
| Li, 2016 | | Decoction | Water | Pinelliae tuber 10g, Codonopsis Pilosulae radix 10g, Zingiberis rhizoma 10g, Scutellariae radix 10g, Coptidis rhizome 8g, Glycyrrhizae radix 6g, Zizyphi fructus 12g | 2 | Not reported | |
| Luo, 2016 | | Decoction | Water | Pinelliae tuber 10g, Codonopsis Pilosulae radix 12g, Zingiberis rhizoma 10g, Scutellariae radix 10g, Coptidis rhizome 8g, Glycyrrhizae radix 6g, Zizyphi fructus 12g | 2 | Not reported | |
| Cai, 2016 | | Decoction | Water | Pinelliae tuber 10g, Codonopsis Pilosulae radix 10g, Zingiberis rhizoma 5g, Scutellariae radix 10g, Coptidis rhizome 5g, Glycyrrhizae radix 5g, 5 Zizyphi fructus | 2 | Not reported | |
| Chen, 2010 | | Decoction | Water | Pinelliae tuber 9g, Codonopsis Pilosulae radix 15g, Zingiberis rhizoma 6g, Scutellariae radix 5g, Glycyrrhizae radix 6g, Zizyphi fructus 10g, Bupleuri radix 6g | 2 | Not reported | |
| Li and An, 2016 | | Decoction | Water | Pinelliae tuber 12g, Codonopsis Pilosulae radix 15g, Zingiberis rhizoma 9g, Scutellariae radix 9g, Coptidis rhizome 9g, Glycyrrhizae radix 6g, Zizyphi fructus 10g | 2 | 300ml | |
| Min, 2009 | | Decoction | Water | Pinelliae tuber 10g, Codonopsis Pilosulae radix 15g, Zingiberis rhizoma 3g, Scutellariae radix 10g, Coptidis rhizome 5g, Glycyrrhizae radix 5g, Zizyphi fructus 10g | 2 | 300ml | |
| Nong, 2017 | | Decoction | Water | Pinelliae tuber 12g, Codonopsis Pilosulae radix 9g, Zingiberis rhizoma 9g, Scutellariae radix 10g, Coptidis rhizome 3g, Glycyrrhizae radix 6g, Zizyphi fructus 10g | 2 | 300ml | |
| Tang, 2015 | | Decoction | Water | Pinelliae tuber 15g, Codonopsis Pilosulae radix 20g, Zingiberis rhizoma 10g, Scutellariae radix 10g, Coptidis rhizome 5g, Glycyrrhizae radix 10g, 4 Zizyphi fructus | 2 | 300ml | |
| Wang, 2007 | | Decoction | Water | Pinelliae tuber 10g, Codonopsis Pilosulae radix 15g, Zingiberis rhizoma 4g, Scutellariae radix 10g, Coptidis rhizome 5g, Glycyrrhizae radix 5g, Zizyphi fructus 10g, Aucklandiae radix 10g, Ponciri fructus immaturus 15g, Curcumae rhizoma 10g | 2 | 300ml | |
| Wang et al*.*,  2012 | | Decoction | Water | Pinelliae tuber 10g, Codonopsis Pilosulae radix 15g, Zingiberis rhizoma 10g, Scutellariae radix 10g, Coptidis rhizome 5g, Glycyrrhizae radix 10g, Zizyphi fructus 4g | 2 | 300-400ml | |
| Wang, 2018 | | Decoction | Water | Pinelliae tuber 9g, Codonopsis Pilosulae radix 6g, Zingiberis rhizoma 6g, Scutellariae radix 9g, Coptidis rhizome 3g, Glycyrrhizae radix 6g, Zizyphi fructus 6g, Ponciri fructus 12g, Curcumae rhizoma 15g, Taraxaci herba 15g | 2 | 400ml | |
| Yi, 2015 | | Decoction | Water | Pinelliae tuber 10g, Pseudostellaria heterophylla root 15g, Zingiberis rhizoma 3g, Scutellariae radix 8g, Coptidis rhizome 6g, Glycyrrhizae radix 6g, Amomi fructus 6g, Magnoliae cortex 10g, Amomi fructus rotundus 10g, Coicis semen 30g | 2 | 300ml | |
| Zhang, 2017 | | Decoction | Water | Pinelliae tuber 12g, Codonopsis Pilosulae radix 9g, Zingiberis rhizoma 9g, Scutellariae radix 10g, Coptidis rhizome 3g, Glycyrrhizae radix 6g, Zizyphi fructus 10g | 3 | 400ml | |
| Zhu and Gu, 2008 | | Decoction | Water | Pinelliae tuber 9g, Codonopsis Pilosulae radix 6g, Zingiberis rhizoma 6g, Scutellariae radix 9g, Coptidis rhizome 3g, Zizyphi fructus 6g, Glycyrrhizae radix 6g, Taraxaci herba 15g, Curcumae rhizoma 15g, Ponciri fructus 12g | 2 | 300ml | |
| Zou, 2015 | | Decoction | Water | Pinelliae tuber 9g, Codonopsis Pilosulae radix 6g, Zingiberis rhizoma 6g, Scutellariae radix 9g, Coptidis rhizome 3g, Zizyphi fructus 6g, Glycyrrhizae radix 6g, Taraxaci herba 15g, Curcumae rhizoma 15g, Ponciri fructus 12g | 3 | Not reported | |
| Shi, 2014 | | Decoction | Water | Pinelliae tuber 10g, Codonopsis Pilosulae radix 15g, Zingiberis rhizoma 4g, Coptidis rhizome 5g, Scutellariae radix 10g, Glycyrrhizae radix 5g, Zizyphi fructus 10g, Aucklandiae radix 10g, Ponciri fructus 10g, Citri Unshius pericarpium 10g | 1 | 100ml | |
| Zhang, 2010 | | Decoction | Water | Pinelliae tuber 10g, Ginseng radix 10g, Zingiberis rhizoma 6g, Scutellariae radix 10g, Coptidis rhizome 6g, Glycyrrhizae radix 10g, Zizyphi fructus 15g | 2 | 400ml | |
| Dong and Chen,  2009 | | Decoction | Water | Pinelliae tuber 9g, Ginseng radix 6g, Zingiberis rhizoma 6g, Scutellariae radix 6g, Glycyrrhizae radix 6g, Zizyphi fructus 4g, Melia toosendan fructus 3g | 2 | 300ml | |
| Fu, 2010 | | Decoction | Water | Pinelliae tuber 10g, Ginseng radix 10g, Zingiberis rhizoma 6g, Scutellariae radix 10g, Coptidis rhizome 6g, Glycyrrhizae radix 10g, Zizyphi fructus 15g | 2 | 400ml | |
| Lang and Cheng,  2015 | | Decoction | Water  After soaking the daily dose in 500ml of cold water for 60 minutes, boil it until it becomes 200ml | Pinelliae tuber 9g, Ginseng radix 6g, Zingiberis rhizoma 6g, Scutellariae radix 6g, Coptidis rhizome 3g, Glycyrrhizae radix 6g, Zizyphi fructus 10g, Ponciri fructus 6g, Bupleuri radix 6g | 2 | 200ml | |
| Liang et al*.*, 2010 | | Decoction | Water After soaking the daily dose in cold water for 60 minutes, boil it | Pinelliae tuber 9g, Ginseng radix 6g, Zingiberis rhizoma 6g, Scutellariae radix 6g, Coptidis rhizome 3g, Glycyrrhizae radix 6g, 12 Zizyphi fructus, Bupleuri radix 6g, Ponciri fructus 6g | 2 | 200ml | |
| Lu, 2018 | | Decoction | Water | Pinelliae tuber 15g, Ginseng radix 9g, Zingiberis rhizoma 10g, Scutellariae radix 9g, Coptidis rhizome 3g, Glycyrrhizae radix 9g, 4 Zizyphi fructus | 2 | 200ml | |
| Su, 2014 | | Decoction | Water | Pinelliae tuber 10g, Codonopsis Pilosulae radix 10g, Zingiberis rhizoma 6g, Scutellariae radix 10g, Coptidis rhizome 6g, Glycyrrhizae radix 6g, 7 Zizyphi fructus | Not reported | Not reported | |
| Yu, 2016 | | Decoction | Water | Pinelliae tuber 10g, Codonopsis Pilosulae radix 10g, Zingiberis rhizoma 5g, Scutellariae radix 10g, Coptidis rhizome 5g, Glycyrrhizae radix 5g, Zizyphi fructus 6g, Bupleuri radix 20g, Paeoniae radix 30g | 2 | 300-400ml | |
| Huang and Long,  2004 | | Decoction | Water | Pinelliae tuber 10g, Codonopsis Pilosulae radix 15g, Zingiberis rhizoma 10g, Scutellariae radix 12g, Coptidis rhizome 6g, 3 pieces of Zingiberis rhizoma, 5 Zizyphi fructus, Atractylodis rhizoma alba 12g, Corydalis tuber 9g, Bupleuri radix 9g, Cyperi rhizoma 9g | 2 | 400ml | |
| Li et al*.*, 2014 | | Decoction | Water | Pinelliae tuber 10g, Ginseng radix 6g, Zingiberis rhizoma 6g, Scutellariae radix 6g, Coptidis rhizome 3g, Glycyrrhizae radix 6g, Zizyphi fructus 5g | 2 | 300-400ml | |
| Zhang et al*.*, 2019 | | Decoction | Water | Pinelliae tuber 15g, Codonopsis Pilosulae radix 15g, Zingiberis rhizoma 6g, Scutellariae radix 20g, Coptidis rhizome 9g, Glycyrrhizae radix 9g, Zizyphi fructus 9g | 2 | | 300-400ml |
| Dang, 2019 | | Decoction | Water | Pinelliae tuber 10g, Zingiberis rhizoma 10g, Coptidis rhizome 4g, Glycyrrhizae radix 10g, 4 Zizyphi fructus, Astragali radix 10g | 2 | | Not reported |
| He and Xie, 2012 | | Decoction | Water | Pinelliae tuber 12g, Codonopsis Pilosulae radix 9g, Zingiberis rhizoma 9g, Scutellariae radix 10g, Coptidis rhizome 3g, Glycyrrhizae radix 6g, 10 Zizyphi fructus | 2 | | Not reported |
| Yin, 2011 | | Decoction | Water | Pinelliae tuber 10g, Codonopsis Pilosulae radix 15g, Zingiberis rhizoma 3g, Scutellariae radix 10g, Coptidis rhizome 6g, Glycyrrhizae radix 6g, Magnoliae cortex 10g | 2 | | Not reported |
| Xie et al*.*, 2011 | | Decoction | Water | Pinelliae tuber 12g, Codonopsis Pilosulae radix 9g, Zingiberis rhizoma 9g, Scutellariae radix 10g, Coptidis rhizome 3g, Glycyrrhizae radix 6g, 10 Zizyphi fructus | 3 | | Not reported |
| Kim, 2017 | | Syrup | Water  Syrup was prepared by the Jeong-woo Pharmaceutical Company Ltd. (Seoul, Korea) and were produced according to Korean Good Manufacturing Practice. | Pinelliae tuber 2.356g, Ginseng radix 1.606g, Zingiberis rhizoma 1g, Scutellariae radix 1.68g, Coptidis rhizome 0.266g, Glycyrrhizae radix 1.464g, Zizyphi fructus 1.024g, Zingiberis rhizoma 0.154g | 2 | | 20g |
| Kim et al*.*, 2021 | | Syrup | Water extraction Syrup was prepared by Jeong-Woo Pharmaceutical Company Ltd. according to Korean Good Clinical Practice guideline. | Pinelliae tuber 2.36g, Ginseng radix 1.6g, Zingiberis rhizoma 1.16g, Scutellariae radix 1.68g, Coptidis rhizome 0.26g, Glycyrrhizae radix 1.46g, Zizyphi fructus 1.02g | 2 | | 20g |
| Park et al*.*, 2013 | | Granules | Water extraction The manufacture was processed according to Korean Good Manufacturing Practice and permitted and regulated by the Korean Food & Drug Administration. | Pinelliae tuber 5.01g, Ginseng radix 3g, Zingiberis rhizoma 2.49g, Scutellariae radix 3g, Coptidis rhizome 1g, Glycyrrhizae radix 3g, Zizyphi fructus 3g | 3 | | 9g |
| Dong, 2018 | | Decoction | Water | Pinelliae tuber 12g, Ginseng radix 9g, Zingiberis rhizoma 9g, Scutellariae radix 9g, Coptidis rhizome 3g, Glycyrrhizae radix 9g, Zizyphi fructus 12g, Angelicae Gigantis radix 10g, , Cinnamomi ramulus 9g | 2 | | Not reported |
| Huang, 2011 | | Decoction | Water | Pinelliae tuber 20g, Ginseng radix 20g, Zingiberis rhizoma 20g, Scutellariae radix 12g, Coptidis rhizome 8g, Glycyrrhizae radix12g, Zizyphi fructus 20g, Crataegi fructus 30g, Massa Medicata fermentata 30g, Fossilia Ossis mastodi 30g, Oyster shell 30g, Arecae semen 20g, Magnoliae cortex 30g | 3 | | 450ml |
| Wang et al*.*, 2019 | | Decoction | Water | Pinelliae tuber 9g, Ginseng radix 6g, Zingiberis rhizoma 6g, Scutellariae radix 6g, Coptidis rhizome 3g, Glycyrrhizae radix 6g, Zizyphi fructus 12g, Bupleuri radix 6g, Ponciri fructus 6g | 2 | | Not reported |
| Yu and Yu, 2017 | | Decoction | Water  Put the daily dose in 1L of water, boil it, and when it becomes 600ml, remove the botanical drugs and boil it until it becomes 300ml | Pinelliae tuber 12g, Ginseng radix 9g, Zingiberis rhizoma 9g, Coptidis rhizome 8g, Glycyrrhizae radix 10g, Zizyphi fructus 12g, Astragali radix 9g | 2 | | 300ml |
| Li and Li, 2004 | | Decoction | Water | Pinelliae tuber 5g, Codonopsis Pilosulae radix 15g, Zingiberis rhizoma 5g, Scutellariae radix 8g, Coptidis rhizome 4g, 5 Zizyphi fructus | 2 | | Not reported |
| Liang et al*.*, 2008 | | Decoction | Water  After soaking the daily dose in cold water for 60 minutes, boil it for 30 minutes | Pinelliae tuber 9g, Ginseng radix 6g, Zingiberis rhizoma 6g, Scutellariae radix 6g, Coptidis rhizome 3g, Glycyrrhizae radix 6g, 12 Zizyphi fructus, Bupleuri radix 6g, Ponciri fructus 6g | 2 | | 200ml |
| Wang, 2001 | | Decoction | Water | Pinelliae tuber 12g, Codonopsis Pilosulae radix 15g, Zingiberis rhizoma 6g, Scutellariae radix 12g, Glycyrrhizae radix 6g, 5 Zizyphi fructus, Atractylodis rhizoma alba 12g, Aucklandiae radix 12g, Melia toosendan fructus 4.5g | 2 | | 300ml |
| Yang F, 2019 | | Decoction | Water | Pinelliae tuber 10g, Codonopsis Pilosulae radix 10g, Zingiberis rhizoma 10g, Scutellariae radix 10g, Coptidis rhizome 10g, Glycyrrhizae radix6g, Bupleuri radix 10g, Ponciri fructus immaturus 10g, Citri Unshius pericarpium 30g, Paeoniae radix 30g, Melia toosendan fructus 10g, Aucklandiae radix 10g, Fritillariae Thunbergii bulbus 15g, Taraxaci herba 10g, Gardeniae fructus 10g, Hordei fructus germinatus 15g | 2 | | Not reported |
| Yang L, 2019 | | Decoction | Water | Pinelliae tuber 15g, Ginseng radix 10g, Zingiberis rhizoma 10g, Scutellariae radix 15g, Coptidis rhizome 6g, Glycyrrhizae radix 10g, Zizyphi fructus 6g, Curcumae Longae rhizoma 10g, Agastachis herba 10g | 2 | | Not reported |
| Tang, 2014 | | Decoction | Water | Pinelliae tuber 10g, Ginseng radix 10g, Zingiberis rhizoma 6g, Scutellariae radix 10g, Coptidis rhizome 6g, Glycyrrhizae radix 10g, Zizyphi fructus 15g | 2 | | 300ml |
| Ding, 2018 | | Decoction | Water | Pinelliae tuber 12g, Ginseng radix 9g, Zingiberis rhizoma 9g, Scutellariae radix 9g, Coptidis rhizome 3g, Glycyrrhizae radix 9g, Zizyphi fructus 12g, Paeoniae radix 10g, Cinnamomi ramulus 9g | 2 | | Not reported |
| Zhao et al*.*, 2013 | | Granules | Not reported Granules were prepared by Tcmages Pharmaceutical Co., Ltd. (Beijing, China). | Pinelliae tuber, Scutellariae radix, Coptidis rhizome, Zingiberis rhizoma, Salviae Miltiorrhizae radix, Glycyrrhizae radix, Magnoliae cortex, Massa Medicata fermentata, Scapharcae seu Tegillarcae concha Dosages by botanical drugs are not reported. | 2 | | Not reported |
| Tian, 2017 | | Granules | Not reported Granules were prepared by Beijing Kangrentang Pharmaceutical Co., Ltd. | Pinelliae tuber 10g, Codonopsis Pilosulae radix 15g, Zingiberis rhizoma 10g, Scutellariae radix 10g, Coptidis rhizome 5g, Glycyrrhizae radix 5g, Magnoliae cortex 10g, Massa Medicata fermentat 15g, Scapharcae seu Tegillarcae concha 30g | 2 | | Not reported |
